# Supplementary material for: Laparoscopically assisted versus open oesophagectomy for patients with oesophageal cancer—the Randomised Oesophagectomy: Minimally Invasive or Open (ROMIO) study: protocol for a randomised controlled trial (RCT)
Source: BMJ Open. 2019 Nov 19;9(11):e030907. doi: 10.1136/bmjopen-2019-030907 (PMC6887040; doi:10.1136/bmjopen-2019-030907)
Supplement: Author's manuscript [file bmjopen-2019-030907.draft_revisions.pdf]

BMJ Open

BMJ Open is committed to open peer review. As part of this commitment we make the peer review history of every article we publish publicly available.

When an article is published we post the peer reviewers' comments and the authors' responses online. We also post the versions of the paper that were used during peer review. These are the versions that the peer review comments apply to.

The versions of the paper that follow are the versions that were submitted during the peer review process. They are not the versions of record or the final published versions. They should not be cited or distributed as the published version of this manuscript.

BMJ Open is an open access journal and the full, final, typeset and author-corrected version of record of the manuscript is available on our site with no access controls, subscription charges or pay-per-view fees (http://bmjopen.bmj.com).

If you have any questions on BMJ Open's open peer review process please email info.bmjopen@bmj.com

BMJ Open

Laparoscopically assisted vs open oesophagectomy for patients with oesophageal cancer – the ROMIO (Randomised Oesophagectomy: Minimally Invasive or Open) study: protocol for a randomized controlled trial (RCT)

Journal:	*BMJ Open*
Manuscript ID	bmjopen-2019-030907
Article Type:	Protocol
Date Submitted by the Author:	11-Apr-2019

Complete List of Authors:	Brierley, Rachel; University of Bristol<br>Gaunt, Daisy; University of Bristol Faculty of Medicine and Dentistry, Bristol Randomised Trials Collaboration & School of Social and Community Medicine; University of Bristol<br>Metcalfe, Chris; University of Bristol, Social Medicine<br>Blazeby, Jane; University of Bristol, Department of Social Medicine<br>Blencowe, Natalie; University of Bristol, Centre for Surgical Research, School of Social and Community Medicine<br>Jepson, Marcus; University of Bristol School of Social and Community Medicine,<br>Berrisford, Richard G.; Derriford Hospital, Upper GI Surgery<br>Avery, Kerry; University of Bristol, School of Social and Community Medicine<br>Hollingworth, William; University of Bristol, School of Social and Community Medicine<br>Rice, Caoimhe; University of Bristol, School of Social and Community Medicine<br>Moure-Fernandez, Aide; University of Bristol<br>Wong, Newton; Department of Cellular Pathology, North Bristol NHS Trust, Southmead Hospital,<br>Nicklin, Joanna; University hospitals Bristol NHS Foundation Trust,<br>Skilton, Anni; University of Bristol<br>Boddy, Alex; Leicester Royal Infirmary<br>Byrne, James; University Hospital Southampton, Division of Surgery, University Hospital Southampton NHS Foundation Trust, Tremona Road, Southampton, Hampshire, SO16 6YD<br>Underwood, Tim; University Hospital Southampton, Division of Surgery, University Hospital Southampton NHS Foundation Trust, Tremona Road, Southampton, Hampshire, SO16 6YD<br>Vohra, Ravi; Nottingham City Hospital<br>Catton, James; Nottingham City Hospital<br>Pursnani, Kish; Royal Preston Hospital<br>Melhado, Rachel; Salford Royal NHS Foundation Trust<br>Alkhaffaf, Bilal; Manchester University NHS Foundation Trust, Department of Oesophago-Gastric Surgery<br>Krysztopik, Richard; Royal United Hospital Bath NHS Trust<br>Lamb, Peter; Royal Infirmary of Edinburgh, General Surgery Department

Culliford, Lucy; University of Bristol, Bristol Heart Institute,
Rogers, Chris; university of bristol, Clinical Science South Bristol
Howes, Ben; University of Bristol, Anesthetics
Chalmers, Katy; University of Bristol
Cousins, Sian; University of Bristol, Population Health Sciences
Elliott, Jackie
Donovan, Jenny; University of Bristol
Heys, Rachael; University of Bristol
Wickens, Robin; University of Bristol
wilkerson, paul; University Hospitals Bristol NHS Foundation Trust
Hollowood, Andrew; University Hospitals Bristol NHS Foundation Trust
streets, christopher; University Hospitals Bristol NHS Foundation Trust
titcomb, dan; University Hospitals Bristol NHS Foundation Trust
humphreys, lee; Derriford Hospital, Upper GI Surgery
wheatley, tim; Derriford Hospital, Upper GI Surgery
sanders, Grant; Derriford Hospital, Upper GI Surgery
ariyarathenam, arun; Derriford Hospital, Upper GI Surgery
kelly, jamie; University Hospital Southampton, Division of Surgery, University Hospital Southampton NHS Foundation Trust, Tremona Road, Southampton, Hampshire, SO16 6YD
noble, fergus; University Hospital Southampton, Division of Surgery, University Hospital Southampton NHS Foundation Trust, Tremona Road, Southampton, Hampshire, SO16 6YD
couper, graeme; Royal Infirmary of Edinburgh, General Surgery Department
skipworth, richard; Royal Infirmary of Edinburgh, General Surgery Department
deans, chris; Royal Infirmary of Edinburgh, General Surgery Department
ubhi, sukhbir; Leicester Royal Infirmary
Williams, R; Leicester Royal Infirmary
bowrey, david; Leicester Royal Infirmary
exon, david; Leicester Royal Infirmary
turner, paul; Royal Preston Hospital
daya shetty, vinutha; Royal Preston Hospital
chaparala, ram; Salford Royal NHS Foundation Trust
akhtar, khurshid; Salford Royal NHS Foundation Trust
farooq, naheed; Salford Royal NHS Foundation Trust
Parsons, Simon; Nottingham University Hospitals NHS Trust, Department of General Surgery
welch, neil; Nottingham City Hospital
Houlihan, Rebecca; University Hospitals Bristol NHS Foundation Trust
Smith, Jo; Derriford Hospital, Upper GI Surgery
Schranz, Rachel; University Hospital Southampton, Division of Surgery, University Hospital Southampton NHS Foundation Trust, Tremona Road, Southampton, Hampshire, SO16 6YD
rea, nicola; Royal Infirmary of Edinburgh, General Surgery Department
cooke, jill; Leicester Royal Infirmary
williams, alexandra; Royal Preston Hospital
hindmarsh, Carolyn; Salford Royal NHS Foundation Trust
maitland, sally; Nottingham City Hospital
howie, lucy; Royal United Hospital Bath NHS Trust
Barham, C; University Hospitals Bristol NHS Foundation Trust, Division of Surgery

Keywords: oesophageal cancer, oesophagectomy, quality of life, randomised controlled trial, minimally invasive oesophagectomy

SCHOLARONE™
Manuscripts

Laparoscopically assisted vs open oesophagectomy for patients with oesophageal cancer – the ROMIO (Randomised Oesophagectomy: Minimally Invasive or Open) study: protocol for a randomized controlled trial (RCT)

Rachel C Brierley[1], Daisy M Gaunt[2], Chris Metcalfe[2], Jane M Blazeby[3,4], Natalie S Blencowe[3,4], Marcus Jepson[3], Richard Berrisford[5], Kerry N L Avery[3], William Hollingworth[6], Caoimhe T Rice[2,6], Aida Moure-Fernandez[6], Newton Wong[7], Joanna Nicklin[4], Anni Skilton[3], Alex Boddy[8], James Byrne[9], Tim Underwood[9], Ravi Vohra[10], James A. Catton[10], Kish Pursnani[11], Rachel Melhado[12], Bilal Alkhaffaf[12], Richard Krysztopik[13], Peter Lamb[14], Lucy Culliford[1], Chris Rogers[1], Benjamin Howes[15], Katy Chalmers[3], Sian Cousins[3], Jackie Elliott, Jenny Donovan[3], Rachael Heys[1], Robin A Wickens[1], Paul Wilkerson[4], Andrew Hollowood[4], Christopher Streets[4], Dan Titcomb[4], M Lee Humphreys[5], Tim Wheatley[5], Grant Sanders[5], Arun Ariyarathenam[5], Jamie Kelly[8], Fergus Noble[8], Graeme Couper[9], Richard J. E. Skipworth[9], Chris Deans[9], Sukhbir Ubhi[10], Rob Williams[10], David Bowrey[10], David Exon[10], Paul Turner[11], Vinutha Shetty[11], Ram Chaparala[12], Khurshid Akhtar[12], Naheed Farooq[12], Simon L. Parsons[13], Neil T Welch[13], Rebecca J Houlihan[4], Joanne Smith[5], Rachel Schranz[8], Nicola Rea[9], Jill Cooke[10], Alexandra Williams[11], Carolyn Hindmarsh[12], Sally Maitland[13], Lucy Howie[14], C Paul Barham[4]

Affiliations:

1. Clinical Trials and Evaluation Unit, Bristol Trials Centre, University of Bristol, University Hospitals Bristol NHS Foundation Trust, Bristol, UK

2. Bristol Randomised Trials Collaboration, Bristol Trials Centre, University of Bristol, Bristol, UK

3. Centre for Surgical Research, Population Health Sciences, Bristol Medical School, Bristol, UK

4. Division of Surgery, University Hospitals Bristol NHS Foundation Trust, Bristol, UK

5. Department of Upper Gastrointestinal Surgery, Derriford Hospital, University Hospitals Plymouth NHS Trust, Plymouth, UK

6. Health Economics Bristol, Bristol Medical School, University of Bristol, Bristol, UK

7. Department of Cellular Pathology, North Bristol NHS Trust, Southmead Hospital, Bristol

8. Leicester Royal Infirmary, University Hospitals of Leicester NHS Trust, Leicester, UK

For peer review only - http://bmjopen.bmj.com/site/about/guidelines.xhtml

9. Southampton General Hospital, University Hospital Southampton NHS Foundation Trust, Southampton, UK

10. City Campus, Nottingham University Hospitals NHS Trust, Nottingham, UK

11. Department of Upper GI surgery, Royal Preston Hospital, Lancashire Teaching Hospitals NHS Foundation Trust, Preston, UK

12. Department of Oesophago-Gastric Surgery, Salford Royal Hospital, Salford Royal NHS foundation Trust, Salford, UK

13. Royal United Hospitals Bath NHS Foundation Trust, Bath, UK

14. Department of Surgery, Royal Infirmary of Edinburgh, NHS Lothian, Edinburgh, UK

15. University Hospitals Bristol NHS Foundation Trust, Bristol, UK

**Corresponding author:*

Dr Rachel C. Brierley,

R.Brierley@bristol.ac.uk,

Clinical Trials and Evaluation Unit Bristol

Bristol Royal Infirmary, Level 7

Queen's Building, Marlborough Street

Bristol

BS2 8HW

0117 342 2987

Number of figures: 2

Number of tables: 0

Word count: 3479

For peer review only - http://bmjopen.bmj.com/site/about/guidelines.xhtml

Abbreviations

AUGIS- Association of Upper Gastro-Intestinal Surgeons
BRTC- Bristol Randomised Trials Collaboration
BTC- Bristol Trials Centre
ConDuCT-II- Collaboration and innovation for Difficult and Complex randomised controlled Trials In Invasive procedures
CTEU- Clinical Trials and Evaluation Unit
CTU- Clinical Trials Unit
EORTC- European Organisation for Research and Treatment of Cancer
HRQL- health related quality of life
HTA- Health Technology Assessment
ICER- Incremental Cost-Effectiveness Ratio
INMB- Incremental Net Monetary Benefit
LAO- laparoscopically-assisted oesophagectomy
MDT – multidisciplinary teamMFI- multidimensional fatigue inventory
MRC- Medical Research Council
NHS- National Health Service
NIHR- National Institute for Health Research
OO- open oesophagectomy
PIL – patient information leaflet
QA- quality assurance
QALY- quality adjusted life years
QLQ- quality of life questionnaire
QRI - QuinteT Recruitment Intervention
RCT- randomised controlled trial
REC- Research Ethics Committee
TMIO- totally minimally invasive oesophagectomy
UK- United Kingdom
UKCRC- UK Clinical Research Collaboration

For peer review only

For peer review only - http://bmjopen.bmj.com/site/about/guidelines.xhtml

Abstract: (up to approx. 250 words)

Introduction: Surgery (oesophagectomy), with neoadjuvant chemo(radio)therapy, is the main curative treatment for patients with oesophageal cancer. Several surgical approaches can be used to remove an oesophageal tumour. The Ivor Lewis (two phase procedure) is usually used in the UK. This can be performed as an open oesophagectomy (OO), a laparoscopically-assisted oesophagectomy (LAO) or a totally minimally invasive oesophagectomy (TMIO). All three are performed in the NHS, with LAO and OO the most common. However, there is limited evidence about which surgical approach is best for patients in terms of survival and post-operative health-related quality of life.

Methods and analysis: We will undertake a UK multicentre randomised controlled trial to compare LAO with OO in adult patients with oesophageal cancer. The primary outcome is patient-reported physical function at 3 and 6 weeks post-operatively and 3 months post-randomisation. Secondary outcomes include: post-operative complications, survival, disease recurrence, other measures of quality of life, spirometry, success of patient blinding and quality assurance measures. A cost-effectiveness analysis will be performed comparing LAO with OO. We will embed a randomised IDEAL phase 2b sub-study to evaluate the safety and evolution of the TMIO procedure and a qualitative recruitment intervention to optimise patient recruitment. We will analyse the primary outcome using a multi-level regression model. Patients will be monitored for up to 3 years after their surgery.

Ethics and dissemination: This study received approval from South-West Frenchay Research Ethics Committee. We will submit the results for publication in a peer-reviewed journal.

Trial registration number: ISRCTN10386621

For peer review only

Article summary

Strengths and limitations of this study

- The ROMIO study will compare laparoscopically-assisted oesophagectomy with open oesophagectomy, which are the procedures most relevant to UK practice.
- We will assess the quality of the surgery, using operative images and pathology.
- The primary outcome (recovery of physical function up to 3 months) considers what matters most to patients about having an oesophagectomy.
- Patients will be blinded to their surgical procedure for 6 days post-operatively (use of large dressings) to achieve an unbiased assessment of pain but it will not be possible to blind patients for the primary outcome.
- ROMIO incorporates a randomised sub-study to collect data on totally minimally invasive oesophagectomy, which is an evolving technique.

For peer review only

For peer review only - http://bmjopen.bmj.com/site/about/guidelines.xhtml

Introduction:

In the UK, about 8900 people are diagnosed with oesophageal cancer each year and the incidence is increasing [1]. Surgical removal of the oesophagus (oesophagectomy), with or without chemo(radio)therapy, is currently the most commonly recommended treatment for patients whose cancer is confined to the oesophagus and the local lymph nodes and who are fit to undergo major surgery. The objective of treatment is a surgical cure but only about 40 to 50% of patients survive for 3 years or more following treatment [1]. The surgical procedure depends on the location and size of the tumour and individual surgeon choice. There are a number of different surgical approaches used in the NHS, but the most commonly used procedure involves removing the bottom part of the oesophagus and part of the top of the stomach (the two-phase Ivor Lewis oesophagectomy). The remaining stomach is fashioned into a tube and brought up into the chest to replace the removed oesophagus.

In the past 10 years there has been an increase in the use of minimally invasive surgical techniques and, according to the latest Association of Upper Gastro-Intestinal Surgeons (AUGIS) audit, 42% of oesophagectomies were performed using Laparoscopically-Assisted Oesophagectomy (LAO) or Totally Minimally Invasive Oesophagectomy (TMIO)[2]. However, it is uncertain whether laparoscopic surgery improves patient recovery after surgery or has any impact on survival.

Observational studies suggest that TMIO achieves the same survival benefit as Open Oesophagectomy (OO) but with better recovery and reduced rates of post-operative pneumonia [3-5], although the apparent faster recovery may be due to the selection of fitter patients for the minimally invasive procedure. To date, seven randomised controlled trials (RCTs) comparing OO with LAO (n=2) or TMIO (n=4) or robot-assisted TMIO (n=1) have been conducted[6-12]. All had modest sample sizes (26-221 patients) and five out of the seven studies were conducted in a single centre (China = 3, Austria = 1, Netherlands = 1). The studies measured short term primary outcomes such as pulmonary infection (n=2)[7, 9],

For peer review only - http://bmjopen.bmj.com/site/about/guidelines.xhtml

post-operative complications (n=4)[6, 10-12] and duration of operation (n=1)[8]. In one RCT, patients were randomised to a surgeon rather than procedure, meaning the treatment effect may be influenced by a difference in skill between surgeons choosing LAO and those choosing OO [11]. All but one of the RCTs[12] were at unclear risk of selection bias, either due to random sequence generation (n=3) or allocation concealment (n=6). The best evidence comes from two multicentre RCTs, the MIRO and TIME trials. MIRO randomised 207 participants in twelve French centres. Patients were randomised to OO (n=104) or LAO (n=103). They compared intra-operative and post-operative complications, classified as grade 2 or above using Clavien-Dindo, within 30 days. There was a lower incidence of complications in those allocated to LAO (36%) compared to those allocated to open oesophagectomy (64%, odds ratio [OR] 0.31, 95% CI 0.18-0.55) However, patients were randomised using opaque envelopes in theatre after a pre-operative laparoscopic investigation [10]. The TIME trial, conducted in five European centres, compared TMIO with OO in 115 patients [7] and reported a 70% reduction in pulmonary infection in the TMIO group in the first 2 weeks post-operatively (relative risk [RR] 0·30, 95% CI 0·12–0·76).[7] However, the TMIO procedure is not well-established in the UK.[13]

We are conducting a large, multicentre RCT (the ROMIO trial) to compare the clinical and cost-effectiveness of LAO vs OO. The trial will provide high quality evidence, relevant to UK practice, of the risks and benefits of LAO, in terms of recovery, health-related quality of life (HRQL), cost and survival. Incorporated into the study are:

- An assessment of the quality of the surgery performed using intra-operative photos of the procedure and pathology reports[14]
- An integrated qualitative QuinteT Recruitment Intervention (QRI) to optimise recruitment[15]

For peer review only - http://bmjopen.bmj.com/site/about/guidelines.xhtml

- A randomised IDEAL 2b sub-study to investigate the safety and technical changes in TMIO[16]

For peer review only

For peer review only - http://bmjopen.bmj.com/site/about/guidelines.xhtml

Methods and analysis:

We have used the SPIRIT reporting guidelines in this protocol paper.[17]

Study design

ROMIO is a multicentre randomised controlled trial (RCT) comparing open oesophagectomy (OO) with laparoscopically-assisted oesophagectomy (LAO) in patients with oesophageal cancer. ROMIO will also include a randomised sub-study in two centres to assess the efficacy of TMIO and review safety data, compared with OO and LAO. The sub-study will also document how the technical aspects of TMIO evolve over time and whether the technique 'stabilises' over the course of ROMIO.

Entry criteria

To ensure comparability between centres and surgeons, centres will only be included if they are undertaking at least 50 oesophagectomies per year and have a minimum of two surgeons participating in ROMIO. Surgeons will be assessed (by submitting two unedited anonymised videos) before they will be permitted to enrol their patients for ROMIO. This quality assurance (QA) measure has been described previously.[14]

Inclusion criteria

We will screen all patients undergoing oesophagectomy (with or without neoadjuvant chemo(radio)therapy) in at least eight UK hospitals for eligibility (see Figure 1). We will include patients who are at least 18 years old, with at least adenocarcinoma or squamous cell cancer of the oesophagus or oesophago-gastric junction, who have been referred for oesophagectomy by the multidisciplinary team after neoadjuvant chemotherapy or chemoradiotherapy (any type). Patients will be included if their tumour is localised (has not spread beyond the local lymph nodes), is more than 5 cm below the crico-pharyngeus (the muscle that keeps the oesophagus shut) and involves less than 4 cm of the stomach wall.

For peer review only - http://bmjopen.bmj.com/site/about/guidelines.xhtml

Patients will only be included if they have been assessed as fit for surgery and are able to provide written informed consent.

Exclusion criteria

Patients will be excluded if they have high-grade dysplasia or if the cancer has spread beyond the oesophagus (T4b) or any stage with M1. All patients must be eligible for either open or minimally invasive surgery and must not be taking part in any other research that would interfere with the ROMIO protocol.

Randomisation

The local research team will take written informed consent from participants. They will then randomise participants up to 2 weeks before their operation using a secure internet-based randomisation system. A computer programme will be used to generate the allocation sequence used for randomisation. Randomisation will be stratified by neoadjuvant treatment and site. Randomisation within blocks of varying size will prevent large imbalances in the number of patients in each treatment group. Participants will be randomised to receive either OO or LAO in a 1:1 ratio (with a varying block size of 6 or 8). In two centres, patients may also be randomised to receive TMIO, in a 1:1:1 ratio (with a varying block size of 6 or 9). The surgical team will be informed of the patient allocation after randomisation and before surgery (see Figure 1).

Trial interventions

The intervention being compared in ROMIO is the surgical approach, i.e. whether the surgeon uses large (OO) or smaller incisions (LAO or TMIO) to perform the operation (see Figure 2). Internally, the operation being performed is expected to be the same, regardless of the surgical approach used. Placement of a feeding jejunostomy or naso-jejunal tube will also at the surgeon’s discretion, as well as the use of intra-abdominal and intra-thoracic

For peer review only - http://bmjopen.bmj.com/site/about/guidelines.xhtml

drains. Details of the surgical technique were established during the feasibility study and as part of the embedded QA study and are intended to be pragmatic.[14, 18] OO will be performed using large incisions in both the abdomen and the chest (see Figure 2); the location and length of incisions are at each surgeon's discretion. LAO will be performed laparoscopically using 5mm and/or 12mm incisions (as many as needed, according to surgeon preference) in the abdomen. One large incision will be made in the chest (see Figure 2). If a feeding jejunostomy tube is placed, this may be performed laparoscopically or by creating an abdominal incision (no bigger than 8cm). In the two centres participating in the sub-study, around 33% of patients will have a TMIO. In this approach, the surgeon will make small incisions in the abdomen and in the chest. For the abdominal part of the procedure, laparoscopic techniques will be used as described above. The surgeon will access the thoracic cavity using 12mm and/or 5mm incisions (as many as needed) and perform the surgery thoracoscopically (see Figure 2).

Procedures to minimise diaphragmatic herniation (where one or more of the abdominal organs moves into the chest) can be performed at the surgeon's discretion. The anastomotic technique and methods to close the incisions are at the surgeon's discretion. Any deviations from the specified procedures must be fully documented and will be reviewed by the study management group.

All surgical interventions will be carried out under general anaesthesia according to local hospital protocols. Patients will receive antibiotics and deep vein thrombosis prophylaxis according to local hospital policies. Co-interventions such as peri-operative analgesia (e.g. epidural anaesthesia or paravertebral catheters) and monitoring (e.g. central or arterial lines) will be permitted according to the preferences of each centre.

Participants have the right to discontinue their part in the study at any time. In addition, the investigator may withdraw the participant from their allocated treatment group if, subsequent

For peer review only - http://bmjopen.bmj.com/site/about/guidelines.xhtml

to randomisation, a clinical reason for not performing the surgical intervention is discovered. Participants withdrawn from their allocated intervention but willing to continue completing follow-up schedules will be encouraged to do so. All discontinuations and withdrawals will be documented.

Primary outcome

The primary outcome is recovery of physical function assessed using the established, validated patient-reported European Organisation for Research and Treatment of Cancer quality of life questionnaire (EORTC-QLQ-C30) at three and six weeks post-surgery and three months after randomisation[19]. This quality of life measure was selected as the key benefits of minimally invasive surgical techniques are the potential for less tissue damage and consequently less pain and a more rapid recovery of function.[20] Patient groups also indicated that quality of life is an outcome that is very important to them.

Secondary outcome measures

Secondary outcomes will assess the efficacy of the OO and LAO in terms of morbidity, survival and safety. Secondary outcomes will include:

Survival

- Overall and disease-free survival for at least 2-years.

Complications

- All-cause short and long-term complications for up to 3 years post-randomisation[21].
- Any complications within 30 days of surgery, as assessed using the Clavien-Dindo System.[22]
- Length of hospital stay (defined as length of primary hospital stay plus readmission within 30 days / length of primary hospital stay plus length of hospital stay if discharged to community hospital).

For peer review only - http://bmjopen.bmj.com/site/about/guidelines.xhtml

- Forced expiratory volume in one second and forced vital capacity measured by spirometry, measured at baseline and days 3 and 6 post-operatively.

Cost-effectiveness

- Incremental net monetary benefit of LAO over OO 2 years after surgery.

Quality of life

- We will measure generic and disease specific aspects of HRQL using the following validated questionnaires at baseline, 6 days, 3 weeks and 6 weeks post-surgery and 3, 6, 9, 12, 18, 24 and, where possible, 36 months post-randomisation:
  - EORTC QLQ-C30[19] – a questionnaire developed to assess the health-related quality of life of cancer patients;
  - EORTC QLQ-OES18[23, 24]– a questionnaire developed to assess the health-related quality of life for oesophageal cancer patients;
  - MFI-20 [25, 26]– a tool widely used to assess fatigue in cancer patients;
  - EuroQOL EQ-5D-5L [27, 28] –a widely used generic quality of life questionnaire.

We will also measure pain pre-operatively and post-operatively at days 3 and 6 using the visual analogue scale (VAS)[29].

Quality assurance

We will assess QA of surgery (reported previously[14]) using:

- Intra-operative photographs will be taken at key stages throughout each procedure and submitted to the study database for on-going monitoring of the operations. Anonymised images will be reviewed and rated by surgical assessors.
- Histopathological measures assessed by pathologists blinded to the treatment allocation, including length of the oesophagus; total counts of nodes – all and malignant (positive) nodes; details of resection margins and pT staging. The slides of 10% of all cases from each centre will be reviewed by the lead pathologist.
- Success of patient blinding during the first six days post-operatively, assessed using the Bang blinding procedure[30]

For peer review only - http://bmjopen.bmj.com/site/about/guidelines.xhtml

Outcome data will be collected onto case report forms (unless questionnaires are specified) and entered onto a study-specific database for data cleaning and analysis.

Data about adverse events will be collected and reported in accordance with Sponsor and regulatory requirements.

Patient and Public Involvement

Patient groups were consulted at the design stage, these feedback from these patient consultations shaped the primary outcome and other aspects of the study. We have patient representatives as grant co-applicants and as independent members of the trial steering committee, in addition to this we will regularly consult patient and public groups about different aspects of the study as it is ongoing. Patients have provided feedback on the burden involved in participating in the research.

Methods used to minimise bias

Patients will be blinded to their treatment allocation by covering all potential incision sites for all surgical approaches (regardless of the actual operation performed) with large dressings for the first 6 days post-operatively. On day 6 patients will be asked to complete a booklet containing all of the quality of life questionnaires (QLQ-C30, OES-18, MFI-20, EQ5D5L) and a pain assessment using the VAS. Success of blinding will be assessed using the Bang Blinding Index.[30] Due to the nature of the study intervention, it is not possible to blind patients for the completion of the quality of life questionnaires at the primary outcome timepoints. However, patients have not had this surgery before so will not have anything to compare it to, furthermore, participants in the study are unlikely to have a strong preference for one approach over another.

Pathologists assessing QA of surgery will also be blinded to the randomised allocation. As the intervention is surgery, there may be variation in surgeon skill or surgical technique. This

For peer review only - http://bmjopen.bmj.com/site/about/guidelines.xhtml

will be managed by stratifying the randomisation by centre. Surgical QA is described in more detail elsewhere.[14]

Loss to follow up will be minimised by maintaining regular contact with patients (by telephone and post) to complete follow-up questionnaires. No additional visits are required for the study.

Sample size

203 participants in each of the LAO and OO groups will allow a minimum clinically important difference of 0.4 standard deviations on the primary outcome to be detected with more than 90% power at the 5% significance level, allowing for 15% of participants not following their allocated procedure, and 10% failing to complete the primary outcome. We anticipate that approximately 40 additional patients will be randomised to TMIO in the sub-study.

Statistical analysis

The results will be reported according to the CONSORT guidelines, including the extension for patient reported outcomes.[31] We will analyse the data according to the intention to treat principle, in that the groups compared will be based on allocated treatment irrespective of the actual operation that the patient had. Participants missing all three assessments contributing to the primary outcome measure will not be included in the primary analysis, but we will use sensitivity analyses to investigate the potential impact of any missing data. We will adjust analyses for treatment centre, whether the participant underwent neoadjuvant chemo(radio)therapy and the baseline value of the outcome under comparison. We will prepare and make publicly available a detailed analysis plan prior to locking the database.

The primary outcome measure (difference between LAO and OO treatment groups) will be the reported difference in mean EORTC-QLQ-C30 scores for recovery of physical function

For peer review only - http://bmjopen.bmj.com/site/about/guidelines.xhtml

(with 95% confidence interval and p-value). The difference in mean scores will be estimated as the co-efficient of a binary variable distinguishing the two treatment groups, in a multi-level regression model, with covariates as detailed. This analysis will be conducted separately for data from the feasibility and main trials, and the two treatment effect estimates pooled as a weighted average. The same approach will be adapted to the assessment of pain during the six days post-operatively and other measures of HRQL.

In addition, we will compare post-operative length of stay and accommodate the skewed distribution of this measure by a log-transformed analysis model presenting ratios of geometric means, 95% confidence interval and p-value.

We will present frequencies of the key treatment complications by treatment allocation. Severity of treatment complications will be compared between allocated treatment groups by scoring each patient according to their most severe Clavien-Dindo category and estimating the difference as an odds ratio using ordered logistic regression.

We will use proportional hazards regression to estimate the treatment difference in overall and disease-free survival. A Kaplan-Meier plot will present survival over time in the OO and LAO groups.

Subgroup analyses

A sub-group analysis will investigate whether the relative effects of OO and LAO differ according to whether a participant underwent neoadjuvant chemotherapy / chemoradiotherapy beforehand.

Analysis of the nested IDEAL 2b study

Data about the TMIO group will be collected and reported separately to the comparison between the OO and LAO groups. We will document the inclusion / exclusion of patients for the TMIO procedure and any reasons for not performing the TMIO operation according to the randomised allocation. We will document the complications of TMIO and perform some

For peer review only - http://bmjopen.bmj.com/site/about/guidelines.xhtml

analyses of safety and adverse events compared with the OO and LAO groups. We will document the evolution of the technical aspects of this procedure according to the IDEAL 2b framework.

Cost-effectiveness analysis

We will convert EQ-5D-5L [18, 19] responses to utilities using the NICE-recommended UK tariff at the time of analysis.[32] These will be combined with survival data to calculate QALYs, adjusted for differences in baseline EQ-5D utility scores.[33] We will estimate theatre costs by collecting detailed information on equipment used and staff time (e.g. surgeons, anaesthetist, scrub nurse). We will collect information on intensive care resource use and re-interventions during the initial hospital stay. We will also collect and analyse data on health care resources used in subsequent inpatient stays, outpatient visits, general practitioner visits and other community health services. We will use nationally available unit costs to value resource use where available.

We will perform the cost-effectiveness analysis on an intention to treat basis from both an NHS perspective, and a wider personal and social care perspective. We will estimate the cost-effectiveness of LAO compared with OO by calculating the Incremental Net Monetary Benefit (INMB) and, if appropriate, the Incremental Cost-Effectiveness Ratio (ICER). We will present uncertainty in these estimates using a cost-effectiveness acceptability curve and/or cost-effectiveness ellipses. We will perform cost analyses at 3 and 24 months post-randomisation. At 24 months, we will discount the cost estimates at the rate recommended by HM Treasury at the time of analysis.[34] We will conduct one-way sensitivity analyses including varying the discount rate. Where appropriate, we will use simple or multiple imputation techniques for missing data.

Ethics and dissemination:

For peer review only - http://bmjopen.bmj.com/site/about/guidelines.xhtml

All study interventions are already routinely used in the NHS. This study has been reviewed and given favourable opinion by the South West - Frenchay Research Ethics Committee on 25th April 2016 (REC, study ref: 184167). We will disseminate the findings by usual academic channels, i.e. presentation at international meetings and peer-reviewed publications. We will write a full report for the funder on the completion of the study and we will provide a lay summary of the results to patient organisations.

Study progress

Recruitment started in October 2016 and we have recruited 277 patients, with an additional 32 TMIO patients (correct on 03 Apr 2019). We have agreed with the funder to continue recruitment until September 2019, beyond the planned completion of recruitment in November 2018. We will also include approximately 120 patients recruited to the feasibility study, having secured permission to continue recruitment to that study whilst the main trial was being set up.

Major changes to the study protocol

Since the first study protocol was approved by the REC (the current version is v7.0, 25 October 2018), we have made the following changes:

- We updated the expected adverse events section to reflect the results of an international consensus paper on standardising reporting of complications of oesophagectomy[21] and to clarify that we will not collect events related to chemotherapy.
- We included information about plans to link with external registries (Intensive Care National Audit Research Centre, Public Health England, Information Services Division) to obtain more detailed data about ROMIO patient care in hospitals, for the purpose of economic analysis and to capture information on acute post-operative complications and recovery.

Figure legends

For peer review only - http://bmjopen.bmj.com/site/about/guidelines.xhtml

Figure 1 CONSORT diagram outline (MDT: multi-disciplinary team; PIL: patient information leaflet; MIO: minimally invasive oesophagectomy; LAO: laparoscopically-assisted oesophagectomy; OO: open oesophagectomy)

Figure 2 Diagrams representing the incisions the surgeon may make for the three different surgical approaches

For peer review only

For peer review only - http://bmjopen.bmj.com/site/about/guidelines.xhtml

References

1. CRUK, C.R. *About oesophageal cancer*. 2018; Available from: https://www.cancerresearchuk.org/about-cancer/oesophageal-cancer/about.
2. Varagunam M, P.M., Sinha S, Cromwell D, Maynard N, Crosby T, Trudgill N, Michalowski J, Napper R, *National Oesophago-Gastric Cancer Audit, 2018.* London: Royal College of Surgeons of England, 2018.
3. Nafteux, P., et al., *Minimally invasive oesophagectomy: a valuable alternative to open oesophagectomy for the treatment of early oesophageal and gastro-oesophageal junction carcinoma.* Eur J Cardiothorac Surg, 2011. **40**(6): p. 1455-63; discussion 1463-4.
4. Sihag, S., et al., *Comparison of perioperative outcomes following open versus minimally invasive Ivor Lewis oesophagectomy at a single, high-volume centre.* Eur J Cardiothorac Surg, 2012. **42**(3): p. 430-7.
5. Tsujimoto, H., et al., *Video-assisted thoracoscopic surgery for esophageal cancer attenuates postoperative systemic responses and pulmonary complications.* Surgery, 2012. **151**(5): p. 667-73.
6. van der Sluis, P.C.M.D.P.M., et al., *Robot-assisted Minimally Invasive Thoracolaparoscopic Esophagectomy Versus Open Transthoracic Esophagectomy for Resectable Esophageal Cancer: A Randomized Controlled Trial.* Annals of Surgery, 2019. **269**(4): p. 621-630.
7. Biere, S.S., et al., *Minimally invasive versus open oesophagectomy for patients with oesophageal cancer: a multicentre, open-label, randomised controlled trial.* Lancet, 2012. **379**(9829): p. 1887-92.
8. Guo, M., et al., *A comparative study of the therapeutic effect in two protocols: video-assisted thoracic surgery combined with laparoscopy versus right open transthoracic esophagectomy for esophageal cancer management.* Chinese-German J Clin Oncol, 2013. **12**(2): p. 68-71.
9. Hong, L., et al., *The Short-Term Outcome of Three-Field Minimally Invasive Esophagectomy for Siewert Type I Esophagogastric Junctional Adenocarcinoma.* Ann Thorac Surg, 2013. **96**: p. 1826–32.
10. Mariette, C., et al., *Hybrid Minimally Invasive Esophagectomy for Esophageal Cancer.* N Engl J Med, 2019. **380**(2): p. 152-162.
11. Ma, G., et al., *Comparison of the short-term clinical outcome between open and minimally invasive esophagectomy by comprehensive complication index.* J Cancer Res Ther, 2018. **14**(4): p. 789-794.
12. Paireder, M., et al., *Morbidity in open versus minimally invasive hybrid esophagectomy (MIOMIE): Long-term results of a randomized controlled clinical study.* Eur Surg, 2018. **50**(6): p. 249-255.
13. Varagunam, M., et al., *National OesophagoGastric Cancer Audit.* 2017.
14. Blencowe, N.S., et al., *Protocol for developing quality assurance measures to use in surgical trials: an example from the ROMIO study.* BMJ Open, 2019. **9**(3): p. e026209.
15. Donovan, J.L., et al., *Optimising recruitment and informed consent in randomised controlled trials: the development and implementation of the Quintet Recruitment Intervention (QRI).* Trials, 2016. **17**(1): p. 283.
16. Hirst, A., et al., *No Surgical Innovation Without Evaluation: Evolution and Further Development of the IDEAL Framework and Recommendations.* Annals of Surgery, 2019. **269**(2): p. 211-220.
17. Chan, A.W., et al., *SPIRIT 2013 statement: defining standard protocol items for clinical trials.* Ann Intern Med, 2013. **158**(3): p. 200-7.
18. Avery, K.N., et al., *The feasibility of a randomized controlled trial of esophagectomy for esophageal cancer--the ROMIO (Randomized Oesophagectomy: Minimally Invasive or Open) study: protocol for a randomized controlled trial.* Trials, 2014. **15**: p. 200.

For peer review only - http://bmjopen.bmj.com/site/about/guidelines.xhtml

19. Aaronson, N.K., et al., *The European Organization for Research and Treatment of Cancer QLQ-C30: a quality-of-life instrument for use in international clinical trials in oncology.* J Natl Cancer Inst, 1993. **85**(5): p. 365-76.
20. Veldkamp, R., et al., *Laparoscopic surgery versus open surgery for colon cancer: short-term outcomes of a randomised trial.* Lancet Oncol, 2005. **6**(7): p. 477-84.
21. Low, D.E., et al., *International Consensus on Standardization of Data Collection for Complications Associated With Esophagectomy: Esophagectomy Complications Consensus Group (ECCG).* Ann Surg, 2015. **262**(2): p. 286-94.
22. Dindo, D., N. Demartines, and P.A. Clavien, *Classification of surgical complications: a new proposal with evaluation in a cohort of 6336 patients and results of a survey.* Ann Surg, 2004. **240**(2): p. 205-13.
23. Blazeby, J.M., et al., *Clinical and psychometric validation of an EORTC questionnaire module, the EORTC QLQ-OES18, to assess quality of life in patients with oesophageal cancer.* Eur J Cancer, 2003. **39**(10): p. 1384-94.
24. Smithers, B.M., et al., *Comparison of the outcomes between open and minimally invasive esophagectomy.* Ann Surg, 2007. **245**(2): p. 232-40.
25. Parameswaran, R., et al., *Health-related quality of life after minimally invasive oesophagectomy.* Br J Surg, 2010. **97**(4): p. 525-31.
26. Smets, E.M., et al., *The Multidimensional Fatigue Inventory (MFI) psychometric qualities of an instrument to assess fatigue.* J Psychosom Res, 1995. **39**(3): p. 315-25.
27. Clinical Outcomes of Surgical Therapy Study Group, *A comparison of laparoscopically assisted and open colectomy for colon cancer.* N Engl J Med, 2004. **350**(20): p. 2050-9.
28. Herdman, M., et al., *Development and preliminary testing of the new five-level version of EQ-5D (EQ-5D-5L).* Qual Life Res, 2011. **20**(10): p. 1727-36.
29. Hauser, K. and D. Walsh, *Visual analogue scales and assessment of quality of life in cancer.* J Support Oncol, 2008. **6**(6): p. 277-82.
30. Bang, H., L. Ni, and C.E. Davis, *Assessment of blinding in clinical trials.* Control Clin Trials, 2004. **25**(2): p. 143-56.
31. Calvert, M., et al., *Reporting of patient-reported outcomes in randomized trials: the CONSORT PRO extension.* JAMA, 2013. **309**(8): p. 814-22.
32. NICE. *Guide to the methods of technology appraisal.* 2013 03 April 2019]; Available from: nice.org.uk/process/pmg9.
33. Manca, A., Hawkins, N, Sculpher, M, *Estimating mean QALYs in trial-based cost-effectiveness analysis: the importance of controlling for baseline utility.* Health Economics, 2005. **14**(5): p. 487-496.
34. Treasury, H., *The Green Book – Central Government Guidance on Appraisal and Evaluation*. 2018.

Author contributions:

All authors critically revised manuscript for important intellectual content and approved the manuscript.

- Rachel C Brierley developed the study design and aims, drafted the protocol and initiated the study, drafted the manuscript and oversaw study conduct and acquisition of data.

For peer review only - http://bmjopen.bmj.com/site/about/guidelines.xhtml

- Daisy M Gaunt developed the study design and aims, drafted the protocol and initiated the study. DMG also oversaw statistical aspects and analyses for the study.
- Chris Metcalfe developed the study concept and obtained grant funding, developed the study design and aims, drafted the protocol and initiated the study. CM also oversaw statistical aspects and analyses for the study.
- Jane M Blazeby developed the study concept and obtained grant funding, developed the study design and aims, drafted the protocol and initiated the study, oversaw the embedded quality assurance study and clinical aspects of the study.
- Natalie S Blencowe developed the study concept and obtained grant funding, developed the study design and aims, drafted the protocol and initiated the study, oversaw the embedded quality assurance study.
- Marcus Jepson developed the study design and aims, drafted the protocol and initiated the study, oversaw the embedded qualitative study.
- Richard Berrisford developed the study concept and obtained grant funding, developed the study design and aims, drafted the protocol and initiated the study. Principal investigator and acquired data for the study.
- Kerry N L Avery developed the study concept and obtained grant funding, developed the study design and aims, drafted the protocol and initiated the study, oversaw patient and public involvement.
- Lucy Culliford developed the study design and aims, drafted the protocol and initiated the study.
- William Hollingworth developed and oversaw health economic aspects of the study.

For peer review only - http://bmjopen.bmj.com/site/about/guidelines.xhtml

- Caoimhe T Rice developed the health economics aspects of the study.
- Newton Wong developed the study concept and obtained grant funding, developed the study design and aims, drafted the protocol and initiated the study and oversaw pathology quality assurance in the study.
- Aida Moure-Fernandez developed the study design and aims, drafted the protocol and initiated the study and developed the health economics aspects of the study.
- Joanna Nicklin developed the study design and aims, drafted the protocol and initiated the study and acquired data for the study.
- Anni Skilton developed the study design and aims, drafted the protocol and initiated the study and oversaw the embedded quality assurance study.
- James Byrne was a Principal investigator and acquired data for the study.
- Tim Underwood was a Principal investigator and acquired data for the study.
- Peter Lamb was a Principal investigator and acquired data for the study.
- Alex Boddy was a Principal investigator and acquired data for the study.
- Kish Pursnani was a Principal investigator and acquired data for the study.
- Rachel Melhado was a Principal investigator and acquired data for the study.
- Bilal Alkhaffaf was a Principal investigator and acquired data for the study.
- Ravi Vohra was a Principal investigator and acquired data for the study.
- James Catton was a Principal investigator and acquired data for the study.

For peer review only - http://bmjopen.bmj.com/site/about/guidelines.xhtml

- Richard Krysztopik was a Principal investigator and acquired data for the study.
- Chris Rogers developed the study concept and obtained grant funding, developed the study design and aims, drafted the protocol and initiated the study.
- Benjamin Howes developed the study concept and obtained grant funding, developed the study design and aims, drafted the protocol and initiated the study.
- Katy Chalmers conducted the systematic review.
- Sian Cousins conducted the systematic review.
- Jackie Elliott developed the study concept and obtained grant funding and contributed to PPI aspects of the study.
- Jenny Donovan developed the study concept and obtained grant funding, developed the study design and aims, drafted the protocol and initiated the study and oversaw the embedded qualitative study.
- Rachael Heyes conducted the study.
- Robin Wickens conducted the study.
- Paul Wilkerson acquired data for the study.
- Andy Hollowood acquired data for the study.
- Christopher Streets acquired data for the study.
- Dan Titcomb acquired data for the study.
- Lee Humphreys acquired data for the study.

For peer review only - http://bmjopen.bmj.com/site/about/guidelines.xhtml

For peer review only

- Tim Wheatley acquired data for the study.
- Grant Sanders acquired data for the study.
- Arun Ariyarathenam acquired data for the study.
- Jamie Kelly acquired data for the study.
- Fergus Noble acquired data for the study.
- Graeme Couper acquired data for the study.
- Richard J. E. Skipworth acquired data for the study.
- Chris Deans acquired data for the study.
- Sukhbir Ubhi acquired data for the study.
- Rob Williams acquired data for the study.
- David Bowrey acquired data for the study.
- David Exon acquired data for the study.
- Paul Turner acquired data for the study.
- Vinutha Shetty acquired data for the study.
- Ram Chaparala acquired data for the study.
- Khurshid Akhtar acquired data for the study.
- Naheed Farooq acquired data for the study.

For peer review only - http://bmjopen.bmj.com/site/about/guidelines.xhtml

- Simon L. Parsons acquired data for the study.
- Neil Welch acquired data for the study.
- Rebecca Houlihan developed the study design and aims, drafted the protocol and initiated the study and acquired data for the study.
- Joanne Smith acquired data for the study.
- Rachel Schranz acquired data for the study.
- Nicola Rea acquired data for the study.
- Jill Cooke acquired data for the study.
- Alexandra Williams acquired data for the study.
- Carolyn Hindmarsh acquired data for the study.
- Sally Maitland acquired data for the study.
- Lucy Howie acquired data for the study.
- C Paul Barham developed the study concept and obtained grant funding, developed the study design and aims, drafted the protocol and initiated the study, oversaw clinical aspects of the study, oversaw the embedded quality assurance study and was Chief Investigator for the study.

Acknowledgements:

We would like to thank the large teams at each hospital who work on the ROMIO study. The hospitals involved with ROMIO are: University Hospitals Bristol NHS Foundation Trust, Bristol, England; University Hospitals Plymouth NHS Trust, Plymouth, England; University Hospital Southampton NHS Foundation Trust, Southampton, England; NHS Lothian, Edinburgh, Scotland; University Hospitals of Leicester NHS Trust, Leicester, England; Lancashire Teaching Hospitals NHS Foundation Trust, Preston, England; Salford Royal NHS Foundation Trust,

For peer review only - http://bmjopen.bmj.com/site/about/guidelines.xhtml

Manchester, England; Nottingham University Hospitals NHS Trust, Nottingham, England; Royal United Hospitals Bath NHS Foundation Trust, Bath, England. We would also like to thank Maria Pufulete, Madeleine Clout and Kate Harris for commenting on the draft manuscript.

This study was sponsored by University Hospitals Bristol NHS Foundation Trust, Research and Innovation, Level 3, UH Bristol Education and Research Centre, Upper Maudlin Street, Bristol BS2 8AE.

The study is overseen by an independent steering committee and an independent data monitoring committee.

Steering Committee membership: Craig Ramsay (Chair, Professor in Health Services Research, University of Aberdeen, Scotland); Tony Ingold (Trustee, Oesophageal Patients Association, England); Heike Grabsch (Professor of Gastrointestinal Pathology, Maastricht University, Netherlands); William Allum (Consultant Upper Gastrointestinal Surgeon, Royal Marsden NHS Foundation Trust, London, England); Richard Hardwick (Consultant Upper Gastrointestinal Surgeon & Lead Clinician for Upper GI Cancer, Cambridge University Hospitals NHS FT, Cambridge, England); Colin Green (Professor of Health Economics, University of Exeter, Exeter, England); Helen Marshall (Principal Statistician, University of Leeds, Leeds, England).

Data Monitoring Committee membership: Judith Bliss (Chair, Professor of Clinical Trials, Institute of Cancer Research, London, England); William Robb (Consultant Upper Gastrointestinal Surgeon, Beaumont Hospital & Royal College of Surgeons in Ireland, Dublin, Ireland); Derek Alderson (President of the Royal College of Surgeons and emeritus Professor of Surgery, University of Birmingham, Birmingham, England).

Funding statement:

This research was funded by the National Institute for Health Research (NIHR) Health Technology Assessment (HTA) programme (project number 14/140/78).

We also received funding support from the Bristol Biomedical Research Centre, the Medical Research Council (MRC) ConDuCT-II (Collaboration and innovation for Difficult and Complex randomised controlled Trials In Invasive procedures) Hub for Trials Methodology Research (MR/K025643/1) and the NIHR Biomedical Research Centre at University Hospitals Bristol NHS Foundation Trust and the University of Bristol.

This study was designed and delivered in collaboration with the Clinical Trials and Evaluation Unit (CTEU) and the Bristol Randomised Trials Collaboration (BRTC), both are UKCRC registered clinical trials units, which are known collectively as the Bristol Trials Centre (BTC), and receive National Institute for Health Research Clinical Trials Unit (CTU) support funding.

Department of Health Disclaimer:

For peer review only - http://bmjopen.bmj.com/site/about/guidelines.xhtml

The views and opinions expressed therein are those of the authors and do not necessarily reflect those of the HTA programme, NIHR, NHS or the Department of Health.

The funder and Sponsors approve any amendments to the study but have no direct involvement in study design; collection, management, analysis, and interpretation of data; writing of the report; and the decision to submit this report for publication.

Competing interests statement

No competing interests are declared

Data Sharing

Data will not be made available for sharing until after publication of the main results of the research. Thereafter, anonymised individual patient data will be made available for secondary research, conditional on assurance from the secondary researcher that the proposed use of the data is compliant with the MRC Policy on Data Preservation and Sharing regarding scientific quality, ethical requirements and value for money. A minimum requirement with respect to scientific quality will be a publicly available pre-specified protocol describing the purpose, methods and analysis of the secondary research, e.g. a protocol for a Cochrane systematic review. The second file containing patient identifiers would be made available for record linkage or a similar purpose, subject to confirmation that the secondary research protocol has been approved by a UK REC or other similar, approved ethics review body.

For peer review only - http://bmjopen.bmj.com/site/about/guidelines.xhtml

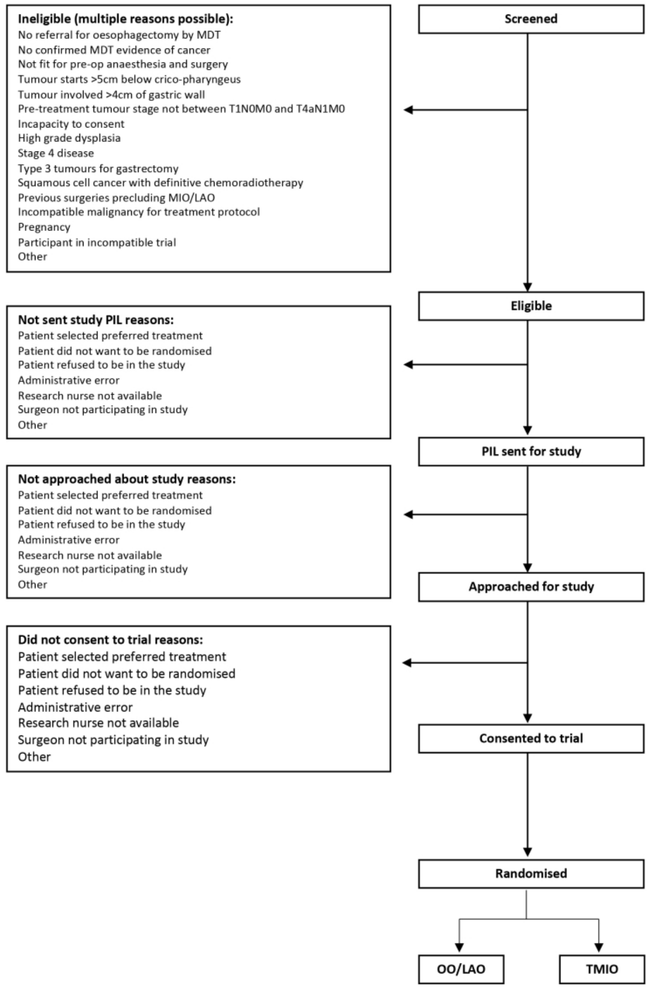

Figure 1 CONSORT diagram outline (MDT: multi-disciplinary team; PIL: patient information leaflet; MIO: minimally invasive oesophagectomy; LAO: laparoscopically-assisted oesophagectomy; OO: open oesophagectomy)

Figure 1 CONSORT diagram outline (MDT: multi-disciplinary team; PIL: patient information leaflet; MIO: minimally invasive oesophagectomy; LAO: laparoscopically-assisted oesophagectomy; OO: open oesophagectomy)

190x275mm (300 x 300 DPI)

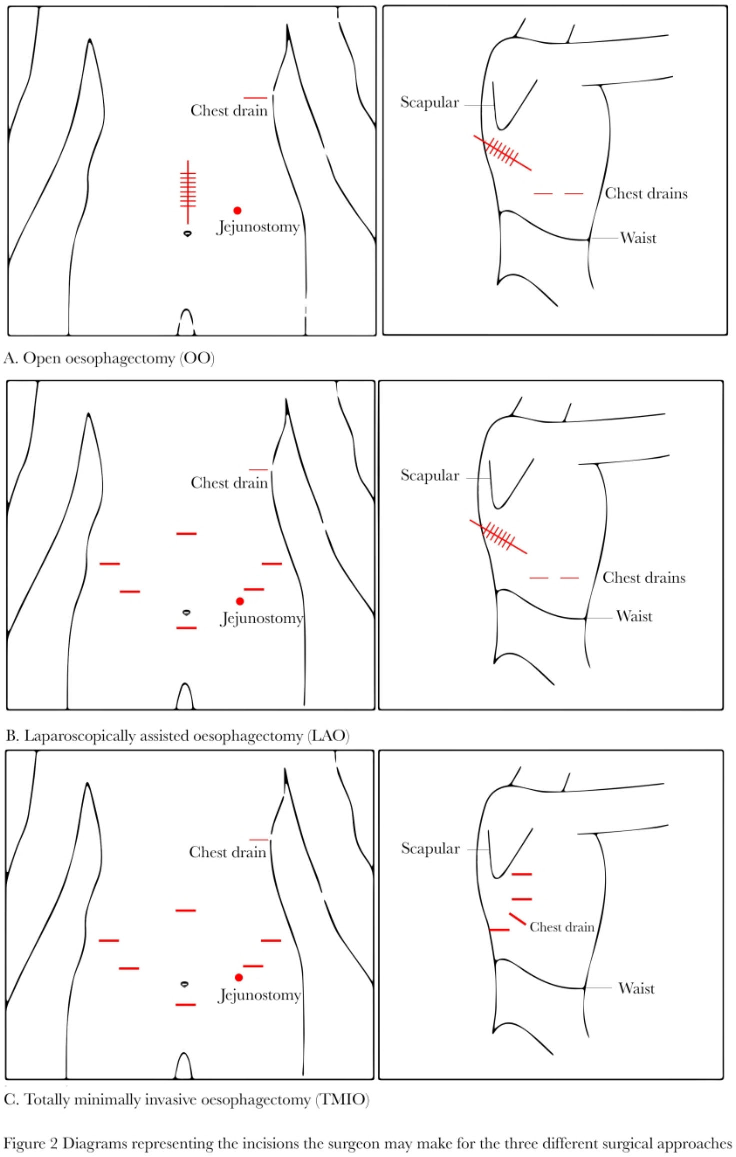

Figure 2 Diagrams representing the incisions the surgeon may make for the three different surgical approaches

293x358mm (300 x 300 DPI)

Reporting checklist for protocol of a clinical trial.

Based on the SPIRIT guidelines.

Instructions to authors

Complete this checklist by entering the page numbers from your manuscript where readers will find each of the items listed below.

Your article may not currently address all the items on the checklist. Please modify your text to include the missing information. If you are certain that an item does not apply, please write "n/a" and provide a short explanation.

Upload your completed checklist as an extra file when you submit to a journal.

In your methods section, say that you used the SPIRIT reporting guidelines, and cite them as:

Chan A-W, Tetzlaff JM, Altman DG, Laupacis A, Gøtzsche PC, Krleža-Jerić K, Hróbjartsson A, Mann H, Dickersin K, Berlin J, Doré C, Parulekar W, Summerskill W, Groves T, Schulz K, Sox H, Rockhold FW, Rennie D, Moher D. SPIRIT 2013 Statement: Defining standard protocol items for clinical trials. Ann Intern Med. 2013;158(3):200-207

		Reporting Item	Page Number
Title	#1	Descriptive title identifying the study design, population, interventions, and, if applicable, trial acronym	1
Trial registration	#2a	Trial identifier and registry name. If not yet registered, name of intended registry	4
Trial registration: data set	#2b	All items from the World Health Organization Trial Registration Data Set	All relevant information is included throughout the paper.
Protocol version	#3	Date and version identifier	17
Funding	#4	Sources and types of financial, material, and other support	21-22
Roles and responsibilities: contributorship	#5a	Names, affiliations, and roles of protocol contributors	1-2, 20-21

For peer review only

Roles and responsibilities: sponsor contact information	#5b	Name and contact information for the trial sponsor	21
Roles and responsibilities: sponsor and funder	#5c	Role of study sponsor and funders, if any, in study design; collection, management, analysis, and interpretation of data; writing of the report; and the decision to submit the report for publication, including whether they will have ultimate authority over any of these activities	22
Roles and responsibilities: committees	#5d	Composition, roles, and responsibilities of the coordinating centre, steering committee, endpoint adjudication committee, data management team, and other individuals or groups overseeing the trial, if applicable (see Item 21a for data monitoring committee)	21
Background and rationale	#6a	Description of research question and justification for undertaking the trial, including summary of relevant studies (published and unpublished) examining benefits and harms for each intervention	6-7
Background and rationale: choice of comparators	#6b	Explanation for choice of comparators	6-7
Objectives	#7	Specific objectives or hypotheses	7
Trial design	#8	Description of trial design including type of trial (eg, parallel group, crossover, factorial, single group), allocation ratio, and framework (eg, superiority, equivalence, non-inferiority, exploratory)	9
Study setting	#9	Description of study settings (eg, community clinic, academic hospital) and list of countries where data will be collected. Reference to where list of study sites can be obtained	21

For peer review only

Eligibility criteria	#10	Inclusion and exclusion criteria for participants. If applicable, eligibility criteria for study centres and individuals who will perform the interventions (eg, surgeons, psychotherapists)	9-10
Interventions: description	#11a	Interventions for each group with sufficient detail to allow replication, including how and when they will be administered	10-11
Interventions: modifications	#11b	Criteria for discontinuing or modifying allocated interventions for a given trial participant (eg, drug dose change in response to harms, participant request, or improving / worsening disease)	11
Interventions: adherance	#11c	Strategies to improve adherence to intervention protocols, and any procedures for monitoring adherence (eg, drug tablet return; laboratory tests)	n/a- ROMIO is a pragmatic trial
Interventions: concomitant care	#11d	Relevant concomitant care and interventions that are permitted or prohibited during the trial	10 (otherwise n/a as ROMIO is a pragmatic trial)
Outcomes	#12	Primary, secondary, and other outcomes, including the specific measurement variable (eg, systolic blood pressure), analysis metric (eg, change from baseline, final value, time to event), method of aggregation (eg, median, proportion), and time point for each outcome. Explanation of the clinical relevance of chosen efficacy and harm outcomes is strongly recommended	12-13
Participant timeline	#13	Time schedule of enrolment, interventions (including any run-ins and washouts), assessments, and visits for participants. A schematic diagram is highly recommended (see Figure)	10, 12-13

For peer review only

Sample size	#14	Estimated number of participants needed to achieve study objectives and how it was determined, including clinical and statistical assumptions supporting any sample size calculations	14
Recruitment	#15	Strategies for achieving adequate participant enrolment to reach target sample size	Not included here
Allocation: sequence generation	#16a	Method of generating the allocation sequence (eg, computer-generated random numbers), and list of any factors for stratification. To reduce predictability of a random sequence, details of any planned restriction (eg, blocking) should be provided in a separate document that is unavailable to those who enrol participants or assign interventions	10
Allocation concealment mechanism	#16b	Mechanism of implementing the allocation sequence (eg, central telephone; sequentially numbered, opaque, sealed envelopes), describing any steps to conceal the sequence until interventions are assigned	10
Allocation: implementation	#16c	Who will generate the allocation sequence, who will enrol participants, and who will assign participants to interventions	10
Blinding (masking)	#17a	Who will be blinded after assignment to interventions (eg, trial participants, care providers, outcome assessors, data analysts), and how	13
Blinding (masking): emergency unblinding	#17b	If blinded, circumstances under which unblinding is permissible, and procedure for revealing a participant's allocated intervention during the trial	N/A –pathologists are blinded. Patients are blinded until a week after surgery, success of patient blinding is assessed.
Data collection plan	#18a	Plans for assessment and collection of outcome, baseline, and other trial data,	12-13

For peer review only

		including any related processes to promote data quality (eg, duplicate measurements, training of assessors) and a description of study instruments (eg, questionnaires, laboratory tests) along with their reliability and validity, if known. Reference to where data collection forms can be found, if not in the protocol	
Data collection plan: retention	#18b	Plans to promote participant retention and complete follow-up, including list of any outcome data to be collected for participants who discontinue or deviate from intervention protocols	11-12, 14
Data management	#19	Plans for data entry, coding, security, and storage, including any related processes to promote data quality (eg, double data entry; range checks for data values). Reference to where details of data management procedures can be found, if not in the protocol	13
Statistics: outcomes	#20a	Statistical methods for analysing primary and secondary outcomes. Reference to where other details of the statistical analysis plan can be found, if not in the protocol	14-15
Statistics: additional analyses	#20b	Methods for any additional analyses (eg, subgroup and adjusted analyses)	15-16
Statistics: analysis population and missing data	#20c	Definition of analysis population relating to protocol non-adherence (eg, as randomised analysis), and any statistical methods to handle missing data (eg, multiple imputation)	14-17
Data monitoring: formal committee	#21a	Composition of data monitoring committee (DMC); summary of its role and reporting structure; statement of whether it is independent from the sponsor and competing interests; and reference to where further details about its charter can be found, if not in	21

For peer review only

		the protocol. Alternatively, an explanation of why a DMC is not needed	
Data monitoring: interim analysis	#21b	Description of any interim analyses and stopping guidelines, including who will have access to these interim results and make the final decision to terminate the trial	n/a – no interim analyses planned
Harms	#22	Plans for collecting, assessing, reporting, and managing solicited and spontaneously reported adverse events and other unintended effects of trial interventions or trial conduct	14
Auditing	#23	Frequency and procedures for auditing trial conduct, if any, and whether the process will be independent from investigators and the sponsor	11
Research ethics approval	#24	Plans for seeking research ethics committee / institutional review board (REC / IRB) approval	17
Protocol amendments	#25	Plans for communicating important protocol modifications (eg, changes to eligibility criteria, outcomes, analyses) to relevant parties (eg, investigators, REC / IRBs, trial participants, trial registries, journals, regulators)	17-18
Consent or assent	#26a	Who will obtain informed consent or assent from potential trial participants or authorised surrogates, and how (see Item 32)	10
Consent or assent: ancillary studies	#26b	Additional consent provisions for collection and use of participant data and biological specimens in ancillary studies, if applicable	n/a
Confidentiality	#27	How personal information about potential and enrolled participants will be collected, shared, and maintained in order to protect confidentiality before, during, and after the trial	21

For peer review only

Declaration of interests	#28	Financial and other competing interests for principal investigators for the overall trial and each study site	22
Data access	#29	Statement of who will have access to the final trial dataset, and disclosure of contractual agreements that limit such access for investigators	22
Ancillary and post trial care	#30	Provisions, if any, for ancillary and post-trial care, and for compensation to those who suffer harm from trial participation	N/A
Dissemination policy: trial results	#31a	Plans for investigators and sponsor to communicate trial results to participants, healthcare professionals, the public, and other relevant groups (eg, via publication, reporting in results databases, or other data sharing arrangements), including any publication restrictions	17
Dissemination policy: authorship	#31b	Authorship eligibility guidelines and any intended use of professional writers	n/a
Dissemination policy: reproducible research	#31c	Plans, if any, for granting public access to the full protocol, participant-level dataset, and statistical code	The protocol is publically available on the NIHR website
Informed consent materials	#32	Model consent form and other related documentation given to participants and authorised surrogates	Available on request
Biological specimens	#33	Plans for collection, laboratory evaluation, and storage of biological specimens for genetic or molecular analysis in the current trial and for future use in ancillary studies, if applicable	N/A

The SPIRIT checklist is distributed under the terms of the Creative Commons Attribution License CC-BY-ND 3.0. This checklist can be completed online using https://www.goodreports.org/, a tool made by the EQUATOR Network in collaboration with Penelope.ai

For peer review only

BMJ Open

Laparoscopically assisted vs open oesophagectomy for patients with oesophageal cancer – the ROMIO (Randomised Oesophagectomy: Minimally Invasive or Open) study: protocol for a randomized controlled trial (RCT)

Journal:	*BMJ Open*
Manuscript ID	bmjopen-2019-030907.R1
Article Type:	Protocol
Date Submitted by the Author:	17-Jun-2019

Complete List of Authors:	Brierley, Rachel; University of Bristol<br>Gaunt, Daisy; University of Bristol Faculty of Medicine and Dentistry, Bristol Randomised Trials Collaboration & School of Social and Community Medicine; University of Bristol<br>Metcalfe, Chris; University of Bristol, Social Medicine<br>Blazeby, Jane; University of Bristol, Department of Social Medicine<br>Blencowe, Natalie; University of Bristol, Centre for Surgical Research, School of Social and Community Medicine<br>Jepson, Marcus; University of Bristol School of Social and Community Medicine,<br>Berrisford, Richard G.; Derriford Hospital, Upper GI Surgery<br>Avery, Kerry; University of Bristol, School of Social and Community Medicine<br>Hollingworth, William; University of Bristol, School of Social and Community Medicine<br>Rice, Caoimhe; University of Bristol, School of Social and Community Medicine<br>Moure-Fernandez, Aide; University of Bristol<br>Wong, Newton; Department of Cellular Pathology, North Bristol NHS Trust, Southmead Hospital,<br>Nicklin, Joanna; University hospitals Bristol NHS Foundation Trust,<br>Skilton, Anni; University of Bristol<br>Boddy, Alex; Leicester Royal Infirmary<br>Byrne, James; University Hospital Southampton, Division of Surgery, University Hospital Southampton NHS Foundation Trust, Tremona Road, Southampton, Hampshire, SO16 6YD<br>Underwood, Tim; University Hospital Southampton, Division of Surgery, University Hospital Southampton NHS Foundation Trust, Tremona Road, Southampton, Hampshire, SO16 6YD<br>Vohra, Ravi; Nottingham City Hospital<br>Catton, James; Nottingham City Hospital<br>Pursnani, Kish; Royal Preston Hospital<br>Melhado, Rachel; Salford Royal NHS Foundation Trust<br>Alkhaffaf, Bilal; Manchester University NHS Foundation Trust, Department of Oesophago-Gastric Surgery<br>Krysztopik, Richard; Royal United Hospital Bath NHS Trust<br>Lamb, Peter; Royal Infirmary of Edinburgh, General Surgery Department

	Culliford, Lucy; University of Bristol, Bristol Heart Institute,<br>Rogers, Chris; university of bristol, Clinical Science South Bristol<br>Howes, Ben; University of Bristol, Anesthetics<br>Chalmers, Katy; University of Bristol<br>Cousins, Sian; University of Bristol, Population Health Sciences<br>Elliott, Jackie<br>Donovan, Jenny; University of Bristol<br>Heys, Rachael; University of Bristol<br>Wickens, Robin; University of Bristol<br>wilkerson, paul; University Hospitals Bristol NHS Foundation Trust<br>Hollowood, Andrew; University Hospitals Bristol NHS Foundation Trust<br>streets, christopher; University Hospitals Bristol NHS Foundation Trust<br>titcomb, dan; University Hospitals Bristol NHS Foundation Trust<br>humphreys, lee; Derriford Hospital, Upper GI Surgery<br>wheatley, tim; Derriford Hospital, Upper GI Surgery<br>sanders, Grant; Derriford Hospital, Upper GI Surgery<br>ariyarathenam, arun; Derriford Hospital, Upper GI Surgery<br>kelly, jamie; University Hospital Southampton, Division of Surgery, University Hospital Southampton NHS Foundation Trust, Tremona Road, Southampton, Hampshire, SO16 6YD<br>noble, fergus; University Hospital Southampton, Division of Surgery, University Hospital Southampton NHS Foundation Trust, Tremona Road, Southampton, Hampshire, SO16 6YD<br>couper, graeme; Royal Infirmary of Edinburgh, General Surgery Department<br>skipworth, richard; Royal Infirmary of Edinburgh, General Surgery Department<br>deans, chris; Royal Infirmary of Edinburgh, General Surgery Department<br>ubhi, sukhbir; Leicester Royal Infirmary<br>Williams, R; Leicester Royal Infirmary<br>bowrey, david; Leicester Royal Infirmary<br>exon, david; Leicester Royal Infirmary<br>turner, paul; Royal Preston Hospital<br>daya shetty, vinutha; Royal Preston Hospital<br>chaparala, ram; Salford Royal NHS Foundation Trust<br>akhtar, khurshid; Salford Royal NHS Foundation Trust<br>farooq, naheed; Salford Royal NHS Foundation Trust<br>Parsons, Simon; Nottingham University Hospitals NHS Trust, Department of General Surgery<br>welch, neil; Nottingham City Hospital<br>Houlihan, Rebecca; University Hospitals Bristol NHS Foundation Trust<br>Smith, Jo; Derriford Hospital, Upper GI Surgery<br>Schranz, Rachel; University Hospital Southampton, Division of Surgery, University Hospital Southampton NHS Foundation Trust, Tremona Road, Southampton, Hampshire, SO16 6YD<br>rea, nicola; Royal Infirmary of Edinburgh, General Surgery Department<br>cooke, jill; Leicester Royal Infirmary<br>williams, alexandra; Royal Preston Hospital<br>hindmarsh, Carolyn; Salford Royal NHS Foundation Trust<br>maitland, sally; Nottingham City Hospital<br>howie, lucy; Royal United Hospital Bath NHS Trust<br>Barham, C; University Hospitals Bristol NHS Foundation Trust, Division of Surgery

**Primary Subject Heading**:	Surgery
Secondary Subject Heading:	Health services research
Keywords:	oesophageal cancer, oesophagectomy, quality of life, randomised controlled trial, minimally invasive oesophagectomy

Laparoscopically assisted vs open oesophagectomy for patients with oesophageal cancer – the ROMIO (Randomised Oesophagectomy: Minimally Invasive or Open) study: protocol for a randomized controlled trial (RCT)

Rachel C Brierley[1], Daisy M Gaunt[2], Chris Metcalfe[2], Jane M Blazeby[3,4], Natalie S Blencowe[3,4], Marcus Jepson[3], Richard Berrisford[5], Kerry N L Avery[3], William Hollingworth[6], Caoimhe T Rice[2,6], Aida Moure-Fernandez[6], Newton Wong[7], Joanna Nicklin[4], Anni Skilton[3], Alex Boddy[8], James Byrne[9], Tim Underwood[9], Ravi Vohra[10], James A. Catton[10], Kish Pursnani[11], Rachel Melhado[12], Bilal Alkhaffaf[12], Richard Krysztopik[13], Peter Lamb[14], Lucy Culliford[1], Chris Rogers[1], Benjamin Howes[15], Katy Chalmers[3], Sian Cousins[3], Jackie Elliott, Jenny Donovan[3], Rachael Heys[1], Robin A Wickens[1], Paul Wilkerson[4], Andrew Hollowood[4], Christopher Streets[4], Dan Titcomb[4], M Lee Humphreys[5], Tim Wheatley[5], Grant Sanders[5], Arun Ariyarathenam[5], Jamie Kelly[8], Fergus Noble[8], Graeme Couper[9], Richard J. E. Skipworth[9], Chris Deans[9], Sukhbir Ubhi[10], Rob Williams[10], David Bowrey[10], David Exon[10], Paul Turner[11], Vinutha Shetty[11], Ram Chaparala[12], Khurshid Akhtar[12], Naheed Farooq[12], Simon L. Parsons[13], Neil T Welch[13], Rebecca J Houlihan[4], Joanne Smith[5], Rachel Schranz[8], Nicola Rea[9], Jill Cooke[10], Alexandra Williams[11], Carolyn Hindmarsh[12], Sally Maitland[13], Lucy Howie[14], C Paul Barham[4]

Affiliations:

1. Clinical Trials and Evaluation Unit, Bristol Trials Centre, University of Bristol, University Hospitals Bristol NHS Foundation Trust, Bristol, UK

2. Bristol Randomised Trials Collaboration, Bristol Trials Centre, University of Bristol, Bristol, UK

3. Centre for Surgical Research, Population Health Sciences, Bristol Medical School, Bristol, UK

4. Division of Surgery, University Hospitals Bristol NHS Foundation Trust, Bristol, UK

5. Department of Upper Gastrointestinal Surgery, Derriford Hospital, University Hospitals Plymouth NHS Trust, Plymouth, UK

6. Health Economics Bristol, Bristol Medical School, University of Bristol, Bristol, UK

7. Department of Cellular Pathology, North Bristol NHS Trust, Southmead Hospital, Bristol

8. Leicester Royal Infirmary, University Hospitals of Leicester NHS Trust, Leicester, UK

For peer review only - http://bmjopen.bmj.com/site/about/guidelines.xhtml

9. Southampton General Hospital, University Hospital Southampton NHS Foundation Trust, Southampton, UK

10. City Campus, Nottingham University Hospitals NHS Trust, Nottingham, UK

11. Department of Upper GI surgery, Royal Preston Hospital, Lancashire Teaching Hospitals NHS Foundation Trust, Preston, UK

12. Department of Oesophago-Gastric Surgery, Salford Royal Hospital, Salford Royal NHS foundation Trust, Salford, UK

13. Royal United Hospitals Bath NHS Foundation Trust, Bath, UK

14. Department of Surgery, Royal Infirmary of Edinburgh, NHS Lothian, Edinburgh, UK

15. University Hospitals Bristol NHS Foundation Trust, Bristol, UK

**Corresponding author:*

Dr Rachel C. Brierley,

R.Brierley@bristol.ac.uk,

Clinical Trials and Evaluation Unit Bristol

Bristol Royal Infirmary, Level 7

Queen’s Building, Marlborough Street

Bristol

BS2 8HW

0117 342 2987

Number of figures: 2

Number of tables: 0

Word count: 3479

For peer review only - http://bmjopen.bmj.com/site/about/guidelines.xhtml

Abbreviations

AUGIS- Association of Upper Gastro-Intestinal Surgeons
BRTC- Bristol Randomised Trials Collaboration
BTC- Bristol Trials Centre
ConDuCT-II- Collaboration and innovation for Difficult and Complex randomised controlled Trials In Invasive procedures
CTEU- Clinical Trials and Evaluation Unit
CTU- Clinical Trials Unit
EORTC- European Organisation for Research and Treatment of Cancer
HRQL- health related quality of life
HTA- Health Technology Assessment
ICER- Incremental Cost-Effectiveness Ratio
INMB- Incremental Net Monetary Benefit
LAO- laparoscopically-assisted oesophagectomy
MDT – multidisciplinary teamMFI- multidimensional fatigue inventory
MRC- Medical Research Council
NHS- National Health Service
NIHR- National Institute for Health Research
OO- open oesophagectomy
PIL – patient information leaflet
QA- quality assurance
QALY- quality adjusted life years
QLQ- quality of life questionnaire
QRI - QuinteT Recruitment Intervention
RCT- randomised controlled trial
REC- Research Ethics Committee
TMIO- totally minimally invasive oesophagectomy
UK- United Kingdom
UKCRC- UK Clinical Research Collaboration

For peer review only

For peer review only - http://bmjopen.bmj.com/site/about/guidelines.xhtml

Abstract: (up to approx. 250 words)

Introduction: Surgery (oesophagectomy), with neoadjuvant chemo(radio)therapy, is the main curative treatment for patients with oesophageal cancer. Several surgical approaches can be used to remove an oesophageal tumour. The Ivor Lewis (two phase procedure) is usually used in the UK. This can be performed as an open oesophagectomy (OO), a laparoscopically-assisted oesophagectomy (LAO) or a totally minimally invasive oesophagectomy (TMIO). All three are performed in the NHS, with LAO and OO the most common. However, there is limited evidence about which surgical approach is best for patients in terms of survival and post-operative health-related quality of life.

Methods and analysis: We will undertake a UK multicentre randomised controlled trial to compare LAO with OO in adult patients with oesophageal cancer. The primary outcome is patient-reported physical function at 3 and 6 weeks post-operatively and 3 months post-randomisation. Secondary outcomes include: post-operative complications, survival, disease recurrence, other measures of quality of life, spirometry, success of patient blinding and quality assurance measures. A cost-effectiveness analysis will be performed comparing LAO with OO. We will embed a randomised IDEAL phase 2b sub-study to evaluate the safety and evolution of the TMIO procedure and a qualitative recruitment intervention to optimise patient recruitment. We will analyse the primary outcome using a multi-level regression model. Patients will be monitored for up to 3 years after their surgery.

Ethics and dissemination: This study received approval from South-West Frenchay Research Ethics Committee. We will submit the results for publication in a peer-reviewed journal.

Trial registration number: ISRCTN10386621

For peer review only

For peer review only - http://bmjopen.bmj.com/site/about/guidelines.xhtml

Article summary

Strengths and limitations of this study

- The ROMIO study will compare laparoscopically-assisted oesophagectomy with open oesophagectomy, which are the procedures most relevant to UK practice.
- We will assess the quality of the surgery, using operative images and pathology.
- The primary outcome (recovery of physical function up to 3 months) considers what matters most to patients about having an oesophagectomy.
- Patients will be blinded to their surgical procedure for 6 days post-operatively (use of large dressings) to achieve an unbiased assessment of pain but it will not be possible to blind patients for the primary outcome.
- ROMIO incorporates a randomised sub-study to collect data on totally minimally invasive oesophagectomy, which is an evolving technique.

For peer review only

For peer review only - http://bmjopen.bmj.com/site/about/guidelines.xhtml

Introduction:

In the UK, about 8900 people are diagnosed with oesophageal cancer each year and the incidence is increasing [1]. Surgical removal of the oesophagus (oesophagectomy), with or without chemo(radio)therapy, is currently the most commonly recommended treatment for patients whose cancer is confined to the oesophagus and the local lymph nodes and who are fit to undergo major surgery. The objective of treatment is a surgical cure but only about 40 to 50% of patients survive for 3 years or more following treatment [1]. The surgical procedure depends on the location and size of the tumour and individual surgeon choice. There are a number of different surgical approaches used in the NHS, but the most commonly used procedure involves removing the bottom part of the oesophagus and part of the top of the stomach (the two-phase Ivor Lewis oesophagectomy). The remaining stomach is fashioned into a tube and brought up into the chest to replace the removed oesophagus.

In the past 10 years there has been an increase in the use of minimally invasive surgical techniques and, according to the latest Association of Upper Gastro-Intestinal Surgeons (AUGIS) audit, 42% of oesophagectomies were performed using Laparoscopically-Assisted Oesophagectomy (LAO) or Totally Minimally Invasive Oesophagectomy (TMIO)[2]. However, it is uncertain whether laparoscopic surgery improves patient recovery after surgery or has any impact on survival.

Observational studies suggest that TMIO achieves the same survival benefit as Open Oesophagectomy (OO) but with better recovery and reduced rates of post-operative pneumonia [3-5], although the apparent faster recovery may be due to the selection of fitter patients for the minimally invasive procedure. To date, seven randomised controlled trials (RCTs) comparing OO with LAO (n=2) or TMIO (n=4) or robot-assisted TMIO (n=1) have been conducted[6-12]. All had modest sample sizes (26-221 patients) and five out of the seven studies were conducted in a single centre (China = 3, Austria = 1, Netherlands = 1). The studies measured short term primary outcomes such as pulmonary infection (n=2)[7, 9],

For peer review only - http://bmjopen.bmj.com/site/about/guidelines.xhtml

post-operative complications (n=4)[6, 10-12] and duration of operation (n=1)[8]. In one RCT, patients were randomised to a surgeon rather than procedure, meaning the treatment effect may be influenced by a difference in skill between surgeons choosing LAO and those choosing OO [11]. All but one of the RCTs[12] were at unclear risk of selection bias, either due to random sequence generation (n=3) or allocation concealment (n=6). The best evidence comes from two multicentre RCTs, the MIRO and TIME trials. MIRO randomised 207 participants in twelve French centres. Patients were randomised to OO (n=104) or LAO (n=103). They compared intra-operative and post-operative complications, classified as grade 2 or above using Clavien-Dindo, within 30 days. There was a lower incidence of complications in those allocated to LAO (36%) compared to those allocated to open oesophagectomy (64%, odds ratio [OR] 0.31, 95% CI 0.18-0.55) However, patients were randomised using opaque envelopes in theatre after a pre-operative laparoscopic investigation [10]. The TIME trial, conducted in five European centres, compared TMIO with OO in 115 patients [7] and reported a 70% reduction in pulmonary infection in the TMIO group in the first 2 weeks post-operatively (relative risk [RR] 0·30, 95% CI 0·12–0·76).[7] However, the TMIO procedure is not well-established in the UK.[13]

We are conducting a large, multicentre RCT (the ROMIO trial) to compare the clinical and cost-effectiveness of LAO vs OO. The trial will provide high quality evidence, relevant to UK practice, of the risks and benefits of LAO, in terms of recovery, health-related quality of life (HRQL), cost and survival. Incorporated into the study are:

- An assessment of the quality of the surgery performed using intra-operative photos of the procedure and pathology reports[14]
- An integrated qualitative QuinteT Recruitment Intervention (QRI) to optimise recruitment[15]

For peer review only - http://bmjopen.bmj.com/site/about/guidelines.xhtml

- A randomised IDEAL 2b sub-study to investigate the safety and technical changes in TMIO[16]

For peer review only

For peer review only - http://bmjopen.bmj.com/site/about/guidelines.xhtml

Methods and analysis:

We have used the SPIRIT reporting guidelines in this protocol paper.[17]

Study design

ROMIO is a multicentre randomised controlled trial (RCT) comparing open oesophagectomy (OO) with laparoscopically-assisted oesophagectomy (LAO) in patients with oesophageal cancer. ROMIO will also include a randomised sub-study in two centres to assess the efficacy of TMIO and review safety data, compared with OO and LAO. The sub-study will also document how the technical aspects of TMIO evolve over time and whether the technique 'stabilises' over the course of ROMIO.

Entry criteria

To ensure comparability between centres and surgeons, centres will only be included if they are undertaking at least 50 oesophagectomies per year and have a minimum of two surgeons participating in ROMIO. Surgeons will be assessed (by electronically submitting two unedited anonymised videos to the ROMIO study imaging team) before they will be permitted to enrol their patients for ROMIO. Further details about this quality assurance (QA) measure has been described previously.[14]

Inclusion criteria

We will screen all patients undergoing oesophagectomy (with or without neoadjuvant chemo(radio)therapy) in at least eight UK hospitals for eligibility (see Figure 1). We will include patients who are at least 18 years old, with at least adenocarcinoma or squamous cell cancer of the oesophagus or oesophago-gastric junction, who have been referred for oesophagectomy by the multidisciplinary team after neoadjuvant chemotherapy or chemoradiotherapy (any type). Patients will be included if their tumour is localised (has not spread beyond the local lymph nodes), is more than 5 cm below the crico-pharyngeus (the muscle that keeps the oesophagus shut) and involves less than 4 cm of the stomach wall.

For peer review only - http://bmjopen.bmj.com/site/about/guidelines.xhtml

Patients will only be included if they have been assessed as fit for surgery and are able to provide written informed consent.

Exclusion criteria

Patients will be excluded if they have high-grade dysplasia or if the cancer has spread beyond the oesophagus (T4b) or any stage with M1. All patients must be eligible for either open or minimally invasive surgery and must not be taking part in any other research that would interfere with the ROMIO protocol.

Randomisation

The local research team will take written informed consent from participants. They will then randomise participants up to 2 weeks before their operation using a secure internet-based randomisation system. A computer programme will be used to generate the allocation sequence used for randomisation. Randomisation will be stratified by neoadjuvant treatment and site. Randomisation within blocks of varying size will prevent large imbalances in the number of patients in each treatment group. Participants will be randomised to receive either OO or LAO in a 1:1 ratio (with a varying block size of 6 or 8). In two centres, patients may also be randomised to receive TMIO, in a 1:1:1 ratio (with a varying block size of 6 or 9). The surgical team will be informed of the patient allocation after randomisation and before surgery (see Figure 1).

Trial interventions

The intervention being compared in ROMIO is the surgical approach, i.e. whether the surgeon uses large (OO) or smaller incisions (LAO or TMIO) to perform the operation (see Figure 2). Internally, the operation being performed is expected to be the same, regardless of the surgical approach used. Placement of a feeding jejunostomy or naso-jejunal tube will also at the surgeon’s discretion, as well as the use of intra-abdominal and intra-thoracic

For peer review only - http://bmjopen.bmj.com/site/about/guidelines.xhtml

drains. Details of the surgical technique were established during the feasibility study and as part of the embedded QA study and are intended to be pragmatic.[14, 18] OO will be performed using large incisions in both the abdomen and the chest (see Figure 2); the location and length of incisions are at each surgeon's discretion. LAO will be performed laparoscopically using 5mm and/or 12mm incisions (as many as needed, according to surgeon preference) in the abdomen. One large incision will be made in the chest (see Figure 2). If a feeding jejunostomy tube is placed, this may be performed laparoscopically or by creating an abdominal incision (no bigger than 8cm). In the two centres participating in the sub-study, around 33% of patients will have a TMIO. In this approach, the surgeon will make small incisions in the abdomen and in the chest. For the abdominal part of the procedure, laparoscopic techniques will be used as described above. The surgeon will access the thoracic cavity using 12mm and/or 5mm incisions (as many as needed) and perform the surgery thoracoscopically (see Figure 2).

Procedures to minimise diaphragmatic herniation (where one or more of the abdominal organs moves into the chest) can be performed at the surgeon's discretion. The anastomotic technique and methods to close the incisions are at the surgeon's discretion. Any deviations from the specified procedures must be fully documented and will be reviewed by the study management group.

All surgical interventions will be carried out under general anaesthesia according to local hospital protocols. Patients will receive antibiotics and deep vein thrombosis prophylaxis according to local hospital policies. Co-interventions such as peri-operative analgesia (e.g. epidural anaesthesia or paravertebral catheters) and monitoring (e.g. central or arterial lines) will be permitted according to the preferences of each centre.

Participants have the right to discontinue their part in the study at any time. In addition, the investigator may withdraw the participant from their allocated treatment group if, subsequent

For peer review only - http://bmjopen.bmj.com/site/about/guidelines.xhtml

to randomisation, a clinical reason for not performing the surgical intervention is discovered. Participants withdrawn from their allocated intervention but willing to continue completing follow-up schedules will be encouraged to do so. All discontinuations and withdrawals will be documented.

Primary outcome

The primary outcome is recovery of physical function assessed using the established, validated patient-reported European Organisation for Research and Treatment of Cancer quality of life questionnaire (EORTC-QLQ-C30) at three and six weeks post-surgery and three months after randomisation[19]. This quality of life measure was selected as the key benefits of minimally invasive surgical techniques are the potential for less tissue damage and consequently less pain and a more rapid recovery of function.[20] Patient groups also indicated that quality of life is an outcome that is very important to them.

Secondary outcome measures

Secondary outcomes will assess the efficacy of the OO and LAO in terms of morbidity, survival and safety. Secondary outcomes will include:

Survival

- Overall and disease-free survival for at least 2-years.

Complications

- All-cause short and long-term complications for up to 3 years post-randomisation[21].
- Any complications within 30 days of surgery, as assessed using the Clavien-Dindo System.[22]
- Length of hospital stay (defined as length of primary hospital stay plus readmission within 30 days / length of primary hospital stay plus length of hospital stay if discharged to community hospital).

For peer review only - http://bmjopen.bmj.com/site/about/guidelines.xhtml

- Forced expiratory volume in one second and forced vital capacity measured by spirometry, measured at baseline and days 3 and 6 post-operatively.

Cost-effectiveness

- Incremental net monetary benefit of LAO over OO 2 years after surgery.

Quality of life

- We will measure generic and disease specific aspects of HRQL using the following validated questionnaires at baseline, 6 days, 3 weeks and 6 weeks post-surgery and 3, 6, 9, 12, 18, 24 and, where possible, 36 months post-randomisation:
  - EORTC QLQ-C30[19] – a questionnaire developed to assess the health-related quality of life of cancer patients;
  - EORTC QLQ-OES18[23, 24]– a questionnaire developed to assess the health-related quality of life for oesophageal cancer patients;
  - MFI-20 [25, 26]– a tool widely used to assess fatigue in cancer patients;
  - EuroQOL EQ-5D-5L [27, 28] –a widely used generic quality of life questionnaire.

We will also measure pain pre-operatively and post-operatively at days 3 and 6 using the visual analogue scale (VAS)[29].

Quality assurance

We will assess QA of surgery (reported previously[14]) using:

- Intra-operative photographs will be taken at key stages throughout each procedure and submitted to the study database for on-going monitoring of the operations. Anonymised images will be reviewed and rated by surgical assessors.
- Histopathological measures assessed by pathologists blinded to the treatment allocation, including length of the oesophagus; total counts of nodes – all and malignant (positive) nodes; details of resection margins and pT staging. The slides of 10% of all cases from each centre will be reviewed by the lead pathologist.
- Success of patient blinding during the first six days post-operatively, assessed using the Bang blinding procedure[30]

For peer review only - http://bmjopen.bmj.com/site/about/guidelines.xhtml

Outcome data will be collected onto case report forms (unless questionnaires are specified) and entered onto a study-specific database for data cleaning and analysis.

Data about adverse events will be collected and reported in accordance with Sponsor and regulatory requirements.

Patient and Public Involvement

Patient groups were consulted at the design stage, these feedback from these patient consultations shaped the primary outcome and other aspects of the study. We have patient representatives as grant co-applicants and as independent members of the trial steering committee, in addition to this we will regularly consult patient and public groups about different aspects of the study as it is ongoing. Patients have provided feedback on the burden involved in participating in the research.

Methods used to minimise bias

Patients will be blinded to their treatment allocation by covering all potential incision sites for all surgical approaches (regardless of the actual operation performed) with large dressings for the first 6 days post-operatively. On day 6 patients will be asked to complete a booklet containing all of the quality of life questionnaires (QLQ-C30, OES-18, MFI-20, EQ5D5L) and a pain assessment using the VAS. Success of blinding will be assessed using the Bang Blinding Index.[30] Due to the nature of the study intervention, it is not possible to blind patients for the completion of the quality of life questionnaires at the primary outcome timepoints. However, patients have not had this surgery before so will not have anything to compare it to, furthermore, participants in the study are unlikely to have a strong preference for one approach over another.

Pathologists assessing QA of surgery will also be blinded to the randomised allocation. As the intervention is surgery, there may be variation in surgeon skill or surgical technique. This

For peer review only

For peer review only - http://bmjopen.bmj.com/site/about/guidelines.xhtml

will be managed by stratifying the randomisation by centre. Surgical QA is described in more detail elsewhere.[14]

Loss to follow up will be minimised by maintaining regular contact with patients (by telephone and post) to complete follow-up questionnaires. No additional visits are required for the study.

Sample size

203 participants in each of the LAO and OO groups will allow a minimum clinically important difference of 0.4 standard deviations on the primary outcome to be detected with more than 90% power at the 5% significance level, allowing for 15% of participants not following their allocated procedure, and 10% failing to complete the primary outcome. We anticipate that approximately 40 additional patients will be randomised to TMIO in the nested IDEAL 2b sub-study to allow us to describe and evaluate changes in technique.

Statistical analysis

The results will be reported according to the CONSORT guidelines, including the extension for patient reported outcomes.[31] We will analyse the data according to the intention to treat principle, in that the groups compared will be based on allocated treatment irrespective of the actual operation that the patient had. Participants missing all three assessments contributing to the primary outcome measure will not be included in the primary analysis, but we will use sensitivity analyses to investigate the potential impact of any missing data. We will adjust analyses for treatment centre, whether the participant underwent neoadjuvant chemo(radio)therapy and the baseline value of the outcome under comparison. We will prepare and make publicly available a detailed analysis plan prior to locking the database.

For peer review only - http://bmjopen.bmj.com/site/about/guidelines.xhtml

For peer review only

The primary outcome measure (difference between LAO and OO treatment groups) will be the reported difference in mean EORTC-QLQ-C30 scores for recovery of physical function (with 95% confidence interval and p-value). The difference in mean scores will be estimated as the co-efficient of a binary variable distinguishing the two treatment groups, in a multi-level regression model, with covariates as detailed. This analysis will be conducted separately for data from the feasibility and main trials, and the two treatment effect estimates pooled as a weighted average. The same approach will be adapted to the assessment of pain during the six days post-operatively and other measures of HRQL.

In addition, we will compare post-operative length of stay and accommodate the skewed distribution of this measure by a log-transformed analysis model presenting ratios of geometric means, 95% confidence interval and p-value.

We will present frequencies of the key treatment complications by treatment allocation. Severity of treatment complications will be compared between allocated treatment groups by scoring each patient according to their most severe Clavien-Dindo category and estimating the difference as an odds ratio using ordered logistic regression.

We will use proportional hazards regression to estimate the treatment difference in overall and disease-free survival. A Kaplan-Meier plot will present survival over time in the OO and LAO groups.

Subgroup analyses

A sub-group analysis will investigate whether the relative effects of OO and LAO differ according to whether a participant underwent neoadjuvant chemotherapy / chemoradiotherapy beforehand.

Analysis of the nested IDEAL 2b study

Data about the TMIO group will be collected and reported separately to the comparison between the OO and LAO groups, these patients will not be included in the main analysis.

For peer review only - http://bmjopen.bmj.com/site/about/guidelines.xhtml

We will document the inclusion / exclusion of patients for the TMIO procedure and any reasons for not performing the TMIO operation according to the randomised allocation. We will document the complications of TMIO and perform some analyses of safety and adverse events compared with the OO and LAO groups. We will document the evolution of the technical aspects of this procedure according to the IDEAL 2b framework.

Cost-effectiveness analysis

We will convert EQ-5D-5L [18, 19] responses to utilities using the NICE-recommended UK tariff at the time of analysis.[32] These will be combined with survival data to calculate QALYs, adjusted for differences in baseline EQ-5D utility scores.[33] We will estimate theatre costs by collecting detailed information on equipment used and staff time (e.g. surgeons, anaesthetist, scrub nurse). We will collect information on intensive care resource use and re-interventions during the initial hospital stay. We will also collect and analyse data on health care resources used in subsequent inpatient stays, outpatient visits, general practitioner visits and other community health services. We will use nationally available unit costs to value resource use where available.

We will perform the cost-effectiveness analysis on an intention to treat basis from both an NHS perspective, and a wider personal and social care perspective. We will estimate the cost-effectiveness of LAO compared with OO by calculating the Incremental Net Monetary Benefit (INMB) and, if appropriate, the Incremental Cost-Effectiveness Ratio (ICER). We will present uncertainty in these estimates using a cost-effectiveness acceptability curve and/or cost-effectiveness ellipses. We will perform cost analyses at 3 and 24 months post-randomisation. At 24 months, we will discount the cost estimates at the rate recommended by HM Treasury at the time of analysis.[34] We will conduct one-way sensitivity analyses including varying the discount rate. Where appropriate, we will use simple or multiple imputation techniques for missing data.

For peer review only - http://bmjopen.bmj.com/site/about/guidelines.xhtml

For peer review only

Ethics and dissemination:

All study interventions are already routinely used in the NHS. This study has been reviewed and given favourable opinion by the South West - Frenchay Research Ethics Committee on 25th April 2016 (REC, study ref: 184167). We will disseminate the findings by usual academic channels, i.e. presentation at international meetings and peer-reviewed publications. We will write a full report for the funder on the completion of the study and we will provide a lay summary of the results to patient organisations.

Study progress

Recruitment started in October 2016 and we have recruited 277 patients, with an additional 32 TMIO patients (correct on 03 Apr 2019). We have agreed with the funder to continue recruitment until September 2019, beyond the planned completion of recruitment in November 2018. We will also include approximately 120 patients recruited to the feasibility study, having secured permission to continue recruitment to that study whilst the main trial was being set up.

Major changes to the study protocol

Since the first study protocol was approved by the REC (the current version is v7.0, 25 October 2018), we have made the following changes:

- We updated the expected adverse events section to reflect the results of an international consensus paper on standardising reporting of complications of oesophagectomy[21] and to clarify that we will not collect events related to chemotherapy.
- We included information about plans to link with external registries (Intensive Care National Audit Research Centre, Public Health England, Information Services Division) to obtain more detailed data about ROMIO patient care in hospitals, for the

For peer review only

For peer review only - http://bmjopen.bmj.com/site/about/guidelines.xhtml

purpose of economic analysis and to capture information on acute post-operative complications and recovery.

Figure legends

Figure 1 CONSORT diagram outline (MDT: multi-disciplinary team; PIL: patient information leaflet; MIO: minimally invasive oesophagectomy; LAO: laparoscopically-assisted oesophagectomy; OO: open oesophagectomy)

Figure 2 Diagrams representing the incisions the surgeon may make for the three different surgical approaches

For peer review only

For peer review only - http://bmjopen.bmj.com/site/about/guidelines.xhtml

References

1. CRUK, C.R. *About oesophageal cancer*. 2018; Available from: https://www.cancerresearchuk.org/about-cancer/oesophageal-cancer/about.
2. Varagunam M, P.M., Sinha S, Cromwell D, Maynard N, Crosby T, Trudgill N, Michalowski J, Napper R, *National Oesophago-Gastric Cancer Audit, 2018.* London: Royal College of Surgeons of England, 2018.
3. Nafteux, P., et al., *Minimally invasive oesophagectomy: a valuable alternative to open oesophagectomy for the treatment of early oesophageal and gastro-oesophageal junction carcinoma.* Eur J Cardiothorac Surg, 2011. **40**(6): p. 1455-63; discussion 1463-4.
4. Sihag, S., et al., *Comparison of perioperative outcomes following open versus minimally invasive Ivor Lewis oesophagectomy at a single, high-volume centre.* Eur J Cardiothorac Surg, 2012. **42**(3): p. 430-7.
5. Tsujimoto, H., et al., *Video-assisted thoracoscopic surgery for esophageal cancer attenuates postoperative systemic responses and pulmonary complications.* Surgery, 2012. **151**(5): p. 667-73.
6. van der Sluis, P.C.M.D.P.M., et al., *Robot-assisted Minimally Invasive Thoracolaparoscopic Esophagectomy Versus Open Transthoracic Esophagectomy for Resectable Esophageal Cancer: A Randomized Controlled Trial.* Annals of Surgery, 2019. **269**(4): p. 621-630.
7. Biere, S.S., et al., *Minimally invasive versus open oesophagectomy for patients with oesophageal cancer: a multicentre, open-label, randomised controlled trial.* Lancet, 2012. **379**(9829): p. 1887-92.
8. Guo, M., et al., *A comparative study of the therapeutic effect in two protocols: video-assisted thoracic surgery combined with laparoscopy versus right open transthoracic esophagectomy for esophageal cancer management.* Chinese-German J Clin Oncol, 2013. **12**(2): p. 68-71.
9. Hong, L., et al., *The Short-Term Outcome of Three-Field Minimally Invasive Esophagectomy for Siewert Type I Esophagogastric Junctional Adenocarcinoma.* Ann Thorac Surg, 2013. **96**: p. 1826–32.
10. Mariette, C., et al., *Hybrid Minimally Invasive Esophagectomy for Esophageal Cancer.* N Engl J Med, 2019. **380**(2): p. 152-162.
11. Ma, G., et al., *Comparison of the short-term clinical outcome between open and minimally invasive esophagectomy by comprehensive complication index.* J Cancer Res Ther, 2018. **14**(4): p. 789-794.
12. Paireder, M., et al., *Morbidity in open versus minimally invasive hybrid esophagectomy (MIOMIE): Long-term results of a randomized controlled clinical study.* Eur Surg, 2018. **50**(6): p. 249-255.
13. Varagunam, M., et al., *National OesophagoGastric Cancer Audit.* 2017.
14. Blencowe, N.S., et al., *Protocol for developing quality assurance measures to use in surgical trials: an example from the ROMIO study.* BMJ Open, 2019. **9**(3): p. e026209.
15. Donovan, J.L., et al., *Optimising recruitment and informed consent in randomised controlled trials: the development and implementation of the Quintet Recruitment Intervention (QRI).* Trials, 2016. **17**(1): p. 283.
16. Hirst, A., et al., *No Surgical Innovation Without Evaluation: Evolution and Further Development of the IDEAL Framework and Recommendations.* Annals of Surgery, 2019. **269**(2): p. 211-220.
17. Chan, A.W., et al., *SPIRIT 2013 statement: defining standard protocol items for clinical trials.* Ann Intern Med, 2013. **158**(3): p. 200-7.
18. Avery, K.N., et al., *The feasibility of a randomized controlled trial of esophagectomy for esophageal cancer--the ROMIO (Randomized Oesophagectomy: Minimally Invasive or Open) study: protocol for a randomized controlled trial.* Trials, 2014. **15**: p. 200.

For peer review only - http://bmjopen.bmj.com/site/about/guidelines.xhtml

19. Aaronson, N.K., et al., *The European Organization for Research and Treatment of Cancer QLQ-C30: a quality-of-life instrument for use in international clinical trials in oncology.* J Natl Cancer Inst, 1993. **85**(5): p. 365-76.
20. Veldkamp, R., et al., *Laparoscopic surgery versus open surgery for colon cancer: short-term outcomes of a randomised trial.* Lancet Oncol, 2005. **6**(7): p. 477-84.
21. Low, D.E., et al., *International Consensus on Standardization of Data Collection for Complications Associated With Esophagectomy: Esophagectomy Complications Consensus Group (ECCG).* Ann Surg, 2015. **262**(2): p. 286-94.
22. Dindo, D., N. Demartines, and P.A. Clavien, *Classification of surgical complications: a new proposal with evaluation in a cohort of 6336 patients and results of a survey.* Ann Surg, 2004. **240**(2): p. 205-13.
23. Blazeby, J.M., et al., *Clinical and psychometric validation of an EORTC questionnaire module, the EORTC QLQ-OES18, to assess quality of life in patients with oesophageal cancer.* Eur J Cancer, 2003. **39**(10): p. 1384-94.
24. Smithers, B.M., et al., *Comparison of the outcomes between open and minimally invasive esophagectomy.* Ann Surg, 2007. **245**(2): p. 232-40.
25. Parameswaran, R., et al., *Health-related quality of life after minimally invasive oesophagectomy.* Br J Surg, 2010. **97**(4): p. 525-31.
26. Smets, E.M., et al., *The Multidimensional Fatigue Inventory (MFI) psychometric qualities of an instrument to assess fatigue.* J Psychosom Res, 1995. **39**(3): p. 315-25.
27. Clinical Outcomes of Surgical Therapy Study Group, *A comparison of laparoscopically assisted and open colectomy for colon cancer.* N Engl J Med, 2004. **350**(20): p. 2050-9.
28. Herdman, M., et al., *Development and preliminary testing of the new five-level version of EQ-5D (EQ-5D-5L).* Qual Life Res, 2011. **20**(10): p. 1727-36.
29. Hauser, K. and D. Walsh, *Visual analogue scales and assessment of quality of life in cancer.* J Support Oncol, 2008. **6**(6): p. 277-82.
30. Bang, H., L. Ni, and C.E. Davis, *Assessment of blinding in clinical trials.* Control Clin Trials, 2004. **25**(2): p. 143-56.
31. Calvert, M., et al., *Reporting of patient-reported outcomes in randomized trials: the CONSORT PRO extension.* JAMA, 2013. **309**(8): p. 814-22.
32. NICE. *Guide to the methods of technology appraisal.* 2013 03 April 2019]; Available from: nice.org.uk/process/pmg9.
33. Manca, A., Hawkins, N, Sculpher, M, *Estimating mean QALYs in trial-based cost-effectiveness analysis: the importance of controlling for baseline utility.* Health Economics, 2005. **14**(5): p. 487-496.
34. Treasury, H., *The Green Book – Central Government Guidance on Appraisal and Evaluation*. 2018.

For peer review only

Author contributions:

All authors critically revised manuscript for important intellectual content and approved the manuscript.

- Rachel C Brierley developed the study design and aims, drafted the protocol and initiated the study, drafted the manuscript and oversaw study conduct and acquisition of data.

For peer review only - http://bmjopen.bmj.com/site/about/guidelines.xhtml

- Daisy M Gaunt developed the study design and aims, drafted the protocol and initiated the study. DMG also oversaw statistical aspects and analyses for the study.

- Chris Metcalfe developed the study concept and obtained grant funding, developed the study design and aims, drafted the protocol and initiated the study. CM also oversaw statistical aspects and analyses for the study.

- Jane M Blazeby developed the study concept and obtained grant funding, developed the study design and aims, drafted the protocol and initiated the study, oversaw the embedded quality assurance study and clinical aspects of the study.

- Natalie S Blencowe developed the study concept and obtained grant funding, developed the study design and aims, drafted the protocol and initiated the study, oversaw the embedded quality assurance study.

- Marcus Jepson developed the study design and aims, drafted the protocol and initiated the study, oversaw the embedded qualitative study.

- Richard Berrisford developed the study concept and obtained grant funding, developed the study design and aims, drafted the protocol and initiated the study. Principal investigator and acquired data for the study.

- Kerry N L Avery developed the study concept and obtained grant funding, developed the study design and aims, drafted the protocol and initiated the study, oversaw patient and public involvement.

- Lucy Culliford developed the study design and aims, drafted the protocol and initiated the study.

- William Hollingworth developed and oversaw health economic aspects of the study.

For peer review only

- Caoimhe T Rice developed the health economics aspects of the study.
- Newton Wong developed the study concept and obtained grant funding, developed the study design and aims, drafted the protocol and initiated the study and oversaw pathology quality assurance in the study.
- Aida Moure-Fernandez developed the study design and aims, drafted the protocol and initiated the study and developed the health economics aspects of the study.
- Joanna Nicklin developed the study design and aims, drafted the protocol and initiated the study and acquired data for the study.
- Anni Skilton developed the study design and aims, drafted the protocol and initiated the study and oversaw the embedded quality assurance study.
- James Byrne was a Principal investigator and acquired data for the study.
- Tim Underwood was a Principal investigator and acquired data for the study.
- Peter Lamb was a Principal investigator and acquired data for the study.
- Alex Boddy was a Principal investigator and acquired data for the study.
- Kish Pursnani was a Principal investigator and acquired data for the study.
- Rachel Melhado was a Principal investigator and acquired data for the study.
- Bilal Alkhaffaf was a Principal investigator and acquired data for the study.
- Ravi Vohra was a Principal investigator and acquired data for the study.
- James Catton was a Principal investigator and acquired data for the study.

For peer review only - http://bmjopen.bmj.com/site/about/guidelines.xhtml

- Richard Krysztopik was a Principal investigator and acquired data for the study.
- Chris Rogers developed the study concept and obtained grant funding, developed the study design and aims, drafted the protocol and initiated the study.
- Benjamin Howes developed the study concept and obtained grant funding, developed the study design and aims, drafted the protocol and initiated the study.
- Katy Chalmers conducted the systematic review.
- Sian Cousins conducted the systematic review.
- Jackie Elliott developed the study concept and obtained grant funding and contributed to PPI aspects of the study.
- Jenny Donovan developed the study concept and obtained grant funding, developed the study design and aims, drafted the protocol and initiated the study and oversaw the embedded qualitative study.
- Rachael Heyes conducted the study.
- Robin Wickens conducted the study.
- Paul Wilkerson acquired data for the study.
- Andy Hollowood acquired data for the study.
- Christopher Streets acquired data for the study.
- Dan Titcomb acquired data for the study.
- Lee Humphreys acquired data for the study.

For peer review only

For peer review only - http://bmjopen.bmj.com/site/about/guidelines.xhtml

- Tim Wheatley acquired data for the study.
- Grant Sanders acquired data for the study.
- Arun Ariyarathenam acquired data for the study.
- Jamie Kelly acquired data for the study.
- Fergus Noble acquired data for the study.
- Graeme Couper acquired data for the study.
- Richard J. E. Skipworth acquired data for the study.
- Chris Deans acquired data for the study.
- Sukhbir Ubhi acquired data for the study.
- Rob Williams acquired data for the study.
- David Bowrey acquired data for the study.
- David Exon acquired data for the study.
- Paul Turner acquired data for the study.
- Vinutha Shetty acquired data for the study.
- Ram Chaparala acquired data for the study.
- Khurshid Akhtar acquired data for the study.
- Naheed Farooq acquired data for the study.

For peer review only

For peer review only - http://bmjopen.bmj.com/site/about/guidelines.xhtml

- Simon L. Parsons acquired data for the study.
- Neil Welch acquired data for the study.
- Rebecca Houlihan developed the study design and aims, drafted the protocol and initiated the study and acquired data for the study.
- Joanne Smith acquired data for the study.
- Rachel Schranz acquired data for the study.
- Nicola Rea acquired data for the study.
- Jill Cooke acquired data for the study.
- Alexandra Williams acquired data for the study.
- Carolyn Hindmarsh acquired data for the study.
- Sally Maitland acquired data for the study.
- Lucy Howie acquired data for the study.
- C Paul Barham developed the study concept and obtained grant funding, developed the study design and aims, drafted the protocol and initiated the study, oversaw clinical aspects of the study, oversaw the embedded quality assurance study and was Chief Investigator for the study.

Acknowledgements:

We would like to thank the large teams at each hospital who work on the ROMIO study. The hospitals involved with ROMIO are: University Hospitals Bristol NHS Foundation Trust, Bristol, England; University Hospitals Plymouth NHS Trust, Plymouth, England; University Hospital Southampton NHS Foundation Trust, Southampton, England; NHS Lothian, Edinburgh, Scotland; University Hospitals of Leicester NHS Trust, Leicester, England; Lancashire Teaching Hospitals NHS Foundation Trust, Preston, England; Salford Royal NHS Foundation Trust,

For peer review only - http://bmjopen.bmj.com/site/about/guidelines.xhtml

For peer review only

Manchester, England; Nottingham University Hospitals NHS Trust, Nottingham, England; Royal United Hospitals Bath NHS Foundation Trust, Bath, England. We would also like to thank Maria Pufulete, Madeleine Clout and Kate Harris for commenting on the draft manuscript.

This study was sponsored by University Hospitals Bristol NHS Foundation Trust, Research and Innovation, Level 3, UH Bristol Education and Research Centre, Upper Maudlin Street, Bristol BS2 8AE.

The study is overseen by an independent steering committee and an independent data monitoring committee.

Steering Committee membership: Craig Ramsay (Chair, Professor in Health Services Research, University of Aberdeen, Scotland); Tony Ingold (Trustee, Oesophageal Patients Association, England); Heike Grabsch (Professor of Gastrointestinal Pathology, Maastricht University, Netherlands); William Allum (Consultant Upper Gastrointestinal Surgeon, Royal Marsden NHS Foundation Trust, London, England); Richard Hardwick (Consultant Upper Gastrointestinal Surgeon & Lead Clinician for Upper GI Cancer, Cambridge University Hospitals NHS FT, Cambridge, England); Colin Green (Professor of Health Economics, University of Exeter, Exeter, England); Helen Marshall (Principal Statistician, University of Leeds, Leeds, England).

Data Monitoring Committee membership: Judith Bliss (Chair, Professor of Clinical Trials, Institute of Cancer Research, London, England); William Robb (Consultant Upper Gastrointestinal Surgeon, Beaumont Hospital & Royal College of Surgeons in Ireland, Dublin, Ireland); Derek Alderson (President of the Royal College of Surgeons and emeritus Professor of Surgery, University of Birmingham, Birmingham, England).

Funding statement:

This research was funded by the National Institute for Health Research (NIHR) Health Technology Assessment (HTA) programme (project number 14/140/78).

We also received funding support from the Bristol Biomedical Research Centre, the Medical Research Council (MRC) ConDuCT-II (Collaboration and innovation for Difficult and Complex randomised controlled Trials In Invasive procedures) Hub for Trials Methodology Research (MR/K025643/1) and the NIHR Biomedical Research Centre at University Hospitals Bristol NHS Foundation Trust and the University of Bristol.

This study was designed and delivered in collaboration with the Clinical Trials and Evaluation Unit (CTEU) and the Bristol Randomised Trials Collaboration (BRTC), both are UKCRC registered clinical trials units, which are known collectively as the Bristol Trials Centre (BTC), and receive National Institute for Health Research Clinical Trials Unit (CTU) support funding.

Department of Health Disclaimer:

For peer review only - http://bmjopen.bmj.com/site/about/guidelines.xhtml

The views and opinions expressed therein are those of the authors and do not necessarily reflect those of the HTA programme, NIHR, NHS or the Department of Health.

The funder and Sponsors approve any amendments to the study but have no direct involvement in study design; collection, management, analysis, and interpretation of data; writing of the report; and the decision to submit this report for publication.

Competing interests statement

No competing interests are declared

Data Sharing

Data will not be made available for sharing until after publication of the main results of the research. Thereafter, anonymised individual patient data will be made available for secondary research, conditional on assurance from the secondary researcher that the proposed use of the data is compliant with the MRC Policy on Data Preservation and Sharing regarding scientific quality, ethical requirements and value for money. A minimum requirement with respect to scientific quality will be a publicly available pre-specified protocol describing the purpose, methods and analysis of the secondary research, e.g. a protocol for a Cochrane systematic review. The second file containing patient identifiers would be made available for record linkage or a similar purpose, subject to confirmation that the secondary research protocol has been approved by a UK REC or other similar, approved ethics review body.

For peer review only

For peer review only - http://bmjopen.bmj.com/site/about/guidelines.xhtml

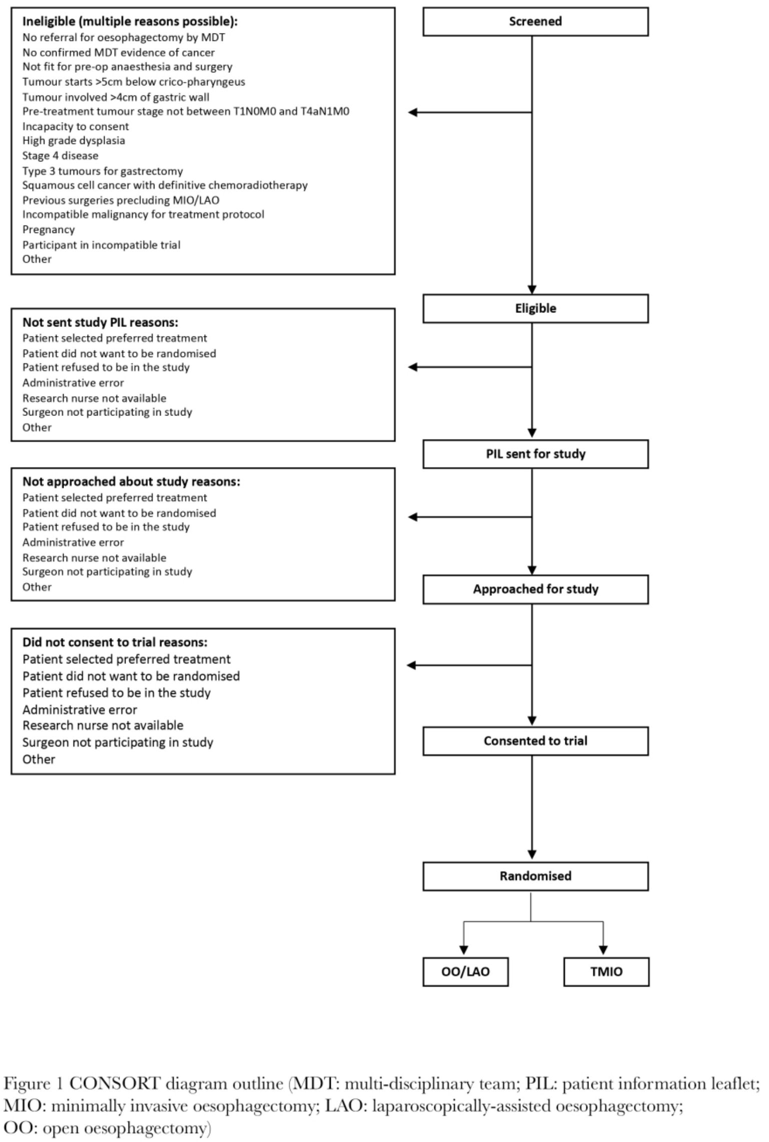

Figure 1 CONSORT diagram outline (MDT: multi-disciplinary team; PIL: patient information leaflet; MIO: minimally invasive oesophagectomy; LAO: laparoscopically-assisted oesophagectomy; OO: open oesophagectomy)

190x275mm (300 x 300 DPI)

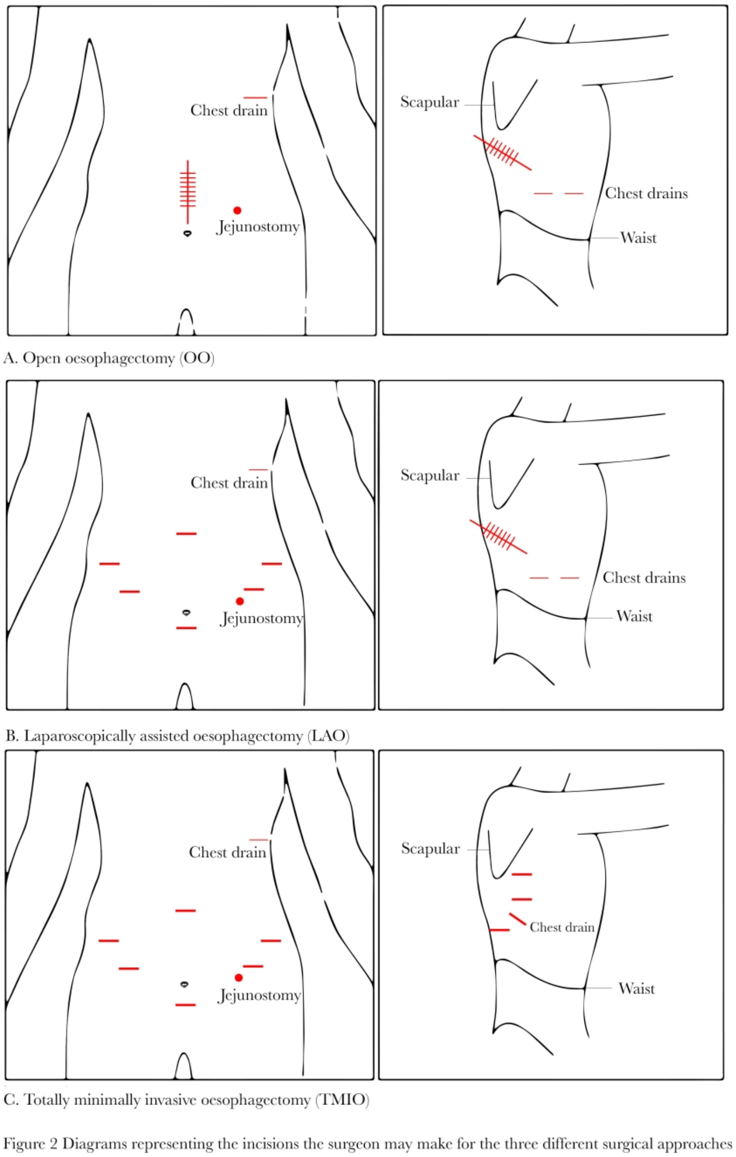

Figure 2 Diagrams representing the incisions the surgeon may make for the three different surgical approaches

293x358mm (300 x 300 DPI)

Reporting checklist for protocol of a clinical trial.

Based on the SPIRIT guidelines.

Instructions to authors

Complete this checklist by entering the page numbers from your manuscript where readers will find each of the items listed below.

Your article may not currently address all the items on the checklist. Please modify your text to include the missing information. If you are certain that an item does not apply, please write "n/a" and provide a short explanation.

Upload your completed checklist as an extra file when you submit to a journal.

In your methods section, say that you used the SPIRIT reporting guidelines, and cite them as:

Chan A-W, Tetzlaff JM, Altman DG, Laupacis A, Gøtzsche PC, Krleža-Jerić K, Hróbjartsson A, Mann H, Dickersin K, Berlin J, Doré C, Parulekar W, Summerskill W, Groves T, Schulz K, Sox H, Rockhold FW, Rennie D, Moher D. SPIRIT 2013 Statement: Defining standard protocol items for clinical trials. Ann Intern Med. 2013;158(3):200-207

		Reporting Item	Page Number
Title	#1	Descriptive title identifying the study design, population, interventions, and, if applicable, trial acronym	1
Trial registration	#2a	Trial identifier and registry name. If not yet registered, name of intended registry	4
Trial registration: data set	#2b	All items from the World Health Organization Trial Registration Data Set	All relevant information is included throughout the paper.
Protocol version	#3	Date and version identifier	17
Funding	#4	Sources and types of financial, material, and other support	21-22
Roles and responsibilities: contributorship	#5a	Names, affiliations, and roles of protocol contributors	1-2, 20-21

For peer review only

Roles and responsibilities: sponsor contact information	#5b	Name and contact information for the trial sponsor	21
Roles and responsibilities: sponsor and funder	#5c	Role of study sponsor and funders, if any, in study design; collection, management, analysis, and interpretation of data; writing of the report; and the decision to submit the report for publication, including whether they will have ultimate authority over any of these activities	22
Roles and responsibilities: committees	#5d	Composition, roles, and responsibilities of the coordinating centre, steering committee, endpoint adjudication committee, data management team, and other individuals or groups overseeing the trial, if applicable (see Item 21a for data monitoring committee)	21
Background and rationale	#6a	Description of research question and justification for undertaking the trial, including summary of relevant studies (published and unpublished) examining benefits and harms for each intervention	6-7
Background and rationale: choice of comparators	#6b	Explanation for choice of comparators	6-7
Objectives	#7	Specific objectives or hypotheses	7
Trial design	#8	Description of trial design including type of trial (eg, parallel group, crossover, factorial, single group), allocation ratio, and framework (eg, superiority, equivalence, non-inferiority, exploratory)	9
Study setting	#9	Description of study settings (eg, community clinic, academic hospital) and list of countries where data will be collected. Reference to where list of study sites can be obtained	21

For peer review only

Eligibility criteria	#10	Inclusion and exclusion criteria for participants. If applicable, eligibility criteria for study centres and individuals who will perform the interventions (eg, surgeons, psychotherapists)	9-10
Interventions: description	#11a	Interventions for each group with sufficient detail to allow replication, including how and when they will be administered	10-11
Interventions: modifications	#11b	Criteria for discontinuing or modifying allocated interventions for a given trial participant (eg, drug dose change in response to harms, participant request, or improving / worsening disease)	11
Interventions: adherance	#11c	Strategies to improve adherence to intervention protocols, and any procedures for monitoring adherence (eg, drug tablet return; laboratory tests)	n/a- ROMIO is a pragmatic trial
Interventions: concomitant care	#11d	Relevant concomitant care and interventions that are permitted or prohibited during the trial	10 (otherwise n/a as ROMIO is a pragmatic trial)
Outcomes	#12	Primary, secondary, and other outcomes, including the specific measurement variable (eg, systolic blood pressure), analysis metric (eg, change from baseline, final value, time to event), method of aggregation (eg, median, proportion), and time point for each outcome. Explanation of the clinical relevance of chosen efficacy and harm outcomes is strongly recommended	12-13
Participant timeline	#13	Time schedule of enrolment, interventions (including any run-ins and washouts), assessments, and visits for participants. A schematic diagram is highly recommended (see Figure)	10, 12-13

For peer review only

Sample size	#14	Estimated number of participants needed to achieve study objectives and how it was determined, including clinical and statistical assumptions supporting any sample size calculations	14
Recruitment	#15	Strategies for achieving adequate participant enrolment to reach target sample size	Not included here
Allocation: sequence generation	#16a	Method of generating the allocation sequence (eg, computer-generated random numbers), and list of any factors for stratification. To reduce predictability of a random sequence, details of any planned restriction (eg, blocking) should be provided in a separate document that is unavailable to those who enrol participants or assign interventions	10
Allocation concealment mechanism	#16b	Mechanism of implementing the allocation sequence (eg, central telephone; sequentially numbered, opaque, sealed envelopes), describing any steps to conceal the sequence until interventions are assigned	10
Allocation: implementation	#16c	Who will generate the allocation sequence, who will enrol participants, and who will assign participants to interventions	10
Blinding (masking)	#17a	Who will be blinded after assignment to interventions (eg, trial participants, care providers, outcome assessors, data analysts), and how	13
Blinding (masking): emergency unblinding	#17b	If blinded, circumstances under which unblinding is permissible, and procedure for revealing a participant's allocated intervention during the trial	N/A –pathologists are blinded. Patients are blinded until a week after surgery, success of patient blinding is assessed.
Data collection plan	#18a	Plans for assessment and collection of outcome, baseline, and other trial data,	12-13

For peer review only

		including any related processes to promote data quality (eg, duplicate measurements, training of assessors) and a description of study instruments (eg, questionnaires, laboratory tests) along with their reliability and validity, if known. Reference to where data collection forms can be found, if not in the protocol	
Data collection plan: retention	#18b	Plans to promote participant retention and complete follow-up, including list of any outcome data to be collected for participants who discontinue or deviate from intervention protocols	11-12, 14
Data management	#19	Plans for data entry, coding, security, and storage, including any related processes to promote data quality (eg, double data entry; range checks for data values). Reference to where details of data management procedures can be found, if not in the protocol	13
Statistics: outcomes	#20a	Statistical methods for analysing primary and secondary outcomes. Reference to where other details of the statistical analysis plan can be found, if not in the protocol	14-15
Statistics: additional analyses	#20b	Methods for any additional analyses (eg, subgroup and adjusted analyses)	15-16
Statistics: analysis population and missing data	#20c	Definition of analysis population relating to protocol non-adherence (eg, as randomised analysis), and any statistical methods to handle missing data (eg, multiple imputation)	14-17
Data monitoring: formal committee	#21a	Composition of data monitoring committee (DMC); summary of its role and reporting structure; statement of whether it is independent from the sponsor and competing interests; and reference to where further details about its charter can be found, if not in	21

For peer review only

		the protocol. Alternatively, an explanation of why a DMC is not needed	
Data monitoring: interim analysis	#21b	Description of any interim analyses and stopping guidelines, including who will have access to these interim results and make the final decision to terminate the trial	n/a – no interim analyses planned
Harms	#22	Plans for collecting, assessing, reporting, and managing solicited and spontaneously reported adverse events and other unintended effects of trial interventions or trial conduct	14
Auditing	#23	Frequency and procedures for auditing trial conduct, if any, and whether the process will be independent from investigators and the sponsor	11
Research ethics approval	#24	Plans for seeking research ethics committee / institutional review board (REC / IRB) approval	17
Protocol amendments	#25	Plans for communicating important protocol modifications (eg, changes to eligibility criteria, outcomes, analyses) to relevant parties (eg, investigators, REC / IRBs, trial participants, trial registries, journals, regulators)	17-18
Consent or assent	#26a	Who will obtain informed consent or assent from potential trial participants or authorised surrogates, and how (see Item 32)	10
Consent or assent: ancillary studies	#26b	Additional consent provisions for collection and use of participant data and biological specimens in ancillary studies, if applicable	n/a
Confidentiality	#27	How personal information about potential and enrolled participants will be collected, shared, and maintained in order to protect confidentiality before, during, and after the trial	21

For peer review only

Declaration of interests	#28	Financial and other competing interests for principal investigators for the overall trial and each study site	22
Data access	#29	Statement of who will have access to the final trial dataset, and disclosure of contractual agreements that limit such access for investigators	22
Ancillary and post trial care	#30	Provisions, if any, for ancillary and post-trial care, and for compensation to those who suffer harm from trial participation	N/A
Dissemination policy: trial results	#31a	Plans for investigators and sponsor to communicate trial results to participants, healthcare professionals, the public, and other relevant groups (eg, via publication, reporting in results databases, or other data sharing arrangements), including any publication restrictions	17
Dissemination policy: authorship	#31b	Authorship eligibility guidelines and any intended use of professional writers	n/a
Dissemination policy: reproducible research	#31c	Plans, if any, for granting public access to the full protocol, participant-level dataset, and statistical code	The protocol is publically available on the NIHR website
Informed consent materials	#32	Model consent form and other related documentation given to participants and authorised surrogates	Available on request
Biological specimens	#33	Plans for collection, laboratory evaluation, and storage of biological specimens for genetic or molecular analysis in the current trial and for future use in ancillary studies, if applicable	N/A

The SPIRIT checklist is distributed under the terms of the Creative Commons Attribution License CC-BY-ND 3.0. This checklist can be completed online using https://www.goodreports.org/, a tool made by the EQUATOR Network in collaboration with Penelope.ai

For peer review only
